# Systematic quantitative analysis of H2A and H2B variants by targeted proteomics

**DOI:** 10.1186/s13072-017-0172-y

**Published:** 2018-01-12

**Authors:** Sara El Kennani, Annie Adrait, Olga Permiakova, Anne-Marie Hesse, Côme Ialy-Radio, Myriam Ferro, Virginie Brun, Julie Cocquet, Jérôme Govin, Delphine Pflieger

**Affiliations:** 1 INSERM U1038, CEA, BIG-BGE, Univ. Grenoble Alpes, Grenoble, France; 2 INSERM U1016, Institut Cochin, CNRS UMR8104, Université Paris Descartes, Sorbonne Paris Cité, Paris, France; 3 CNRS, FR CNRS 3425, Biosciences and Biotechnology Institute of Grenoble, Grenoble, France

**Keywords:** Histone variants, Chromatin, Proteomics, Targeted proteomics, SRM, PRM, Spermatogenesis

## Abstract

**Background:**

Histones organize DNA into chromatin through a variety of processes. Among them, a vast diversity of histone variants can be incorporated into chromatin and finely modulate its organization and functionality. Classically, the study of histone variants has largely relied on antibody-based assays. However, antibodies have a limited efficiency to discriminate between highly similar histone variants.

**Results:**

In this study, we established a mass spectrometry-based analysis to address this challenge. We developed a targeted proteomics method, using selected reaction monitoring or parallel reaction monitoring, to quantify a maximum number of histone variants in a single multiplexed assay, even when histones are present in a crude extract. This strategy was developed on H2A and H2B variants, using 55 peptides corresponding to 25 different histone sequences, among which a few differ by a single amino acid. The methodology was then applied to mouse testis extracts in which almost all histone variants are expressed. It confirmed the abundance profiles of several testis-specific histones during successive stages of spermatogenesis and the existence of predicted H2A.L.1 isoforms. This methodology was also used to explore the over-expression pattern of H2A.L.1 isoforms in a mouse model of male infertility.

**Conclusions:**

Our results demonstrate that targeted proteomics is a powerful method to quantify highly similar histone variants and isoforms. The developed method can be easily transposed to the study of human histone variants, whose abundance can be deregulated in various diseases.

**Electronic supplementary material:**

The online version of this article (10.1186/s13072-017-0172-y) contains supplementary material, which is available to authorized users.

## Background

The basic unit of chromatin is the nucleosome, an octamer of four core histones, H2A, H2B, H3, and H4. The assembly of eight histone molecules into a nucleosome is mediated by the histone fold, a central globular domain, to form a core particle around which 147 base pairs of DNA wrap [1].

The tight control of nucleosomal organization is critical for many cellular processes, such as the regulation of transcription, DNA replication, and DNA repair [2, 3]. A vast diversity of regulatory mechanisms are involved in these nuclear processes, such as DNA methylation, histone modifications, and chromatin remodeling by protein complexes and noncoding regulatory RNAs [4–7]. The existence of histone variants adds a level of complexity to these mechanisms. Indeed, 83 histone variants (including splicing isoforms) have been identified in mouse for histones H2A, H2B, and H3 that largely expand the diversity of nucleosomal actors involved in chromatin signaling pathways [8].

Antibodies are routinely used to explore the functional roles of histone variants. Many of them are now commercially available and widely utilized by research groups for the quantification and visualization of histones by classical biochemical approaches, such as western blots, immunofluorescence, and immunoprecipitation. These bio-reagents have notably the advantage of being highly sensitive when combined with secondary detection methodologies. They thus allowed monitoring the abundance of histone variants in several cellular or pathological contexts [9–14].

However, antibody-based techniques show limitations regarding specificity and throughput. For instance, H2A histone variants exhibit extremely high-sequence similarity that can go beyond 90% for H2A.L.1 variants or canonical H2A and H2A.X histones. In addition, histones are notoriously decorated by a multitude of post-translational modifications (PTMs), which further complicates the generation of antibodies [15]. Finally, lot-to-lot variations of antibodies can result in a lack of reproducibility [16, 17].

Mass spectrometry (MS) has now become a powerful analytical strategy to qualitatively and quantitatively study proteins and their PTMs. Different MS-based approaches have been implemented to characterize histones, by analyzing polypeptides of different sizes: bottom-up analysis of smaller peptides [18–22], middle-down analysis of larger peptides, typically spanning the about 50 N-terminal residues of H3 or H4 [21, 23], and top-down analysis of intact proteins [22, 24–27]. Discovery proteomics aims at identifying and quantifying a maximum number of proteins in a biological sample. In a bottom-up approach, proteins extracted from biological samples are processed into peptides, usually with the protease trypsin which cleaves peptidic bonds after the basic amino acids lysine and arginine. The resulting peptides are then separated by liquid chromatography (LC) before their on-line analysis by the mass spectrometer. Following measurement of their accurate mass-to-charge (m/z) ratios, peptides are fragmented to obtain amino acid sequence information. In a discovery-based proteomics approach, the peptides giving rise to the most intense signals in MS are automatically selected for fragmentation by MS/MS. The acquired MS/MS spectra are finally matched to theoretical fragment spectra to determine the most likely peptide sequences. Such approaches are very powerful to characterize complex samples. Yet, in spite of the increased sensitivity and dynamic range of recent mass spectrometry instruments, lower abundance proteins may still be hidden by the major protein components in the sample.

**Fig. 1:**
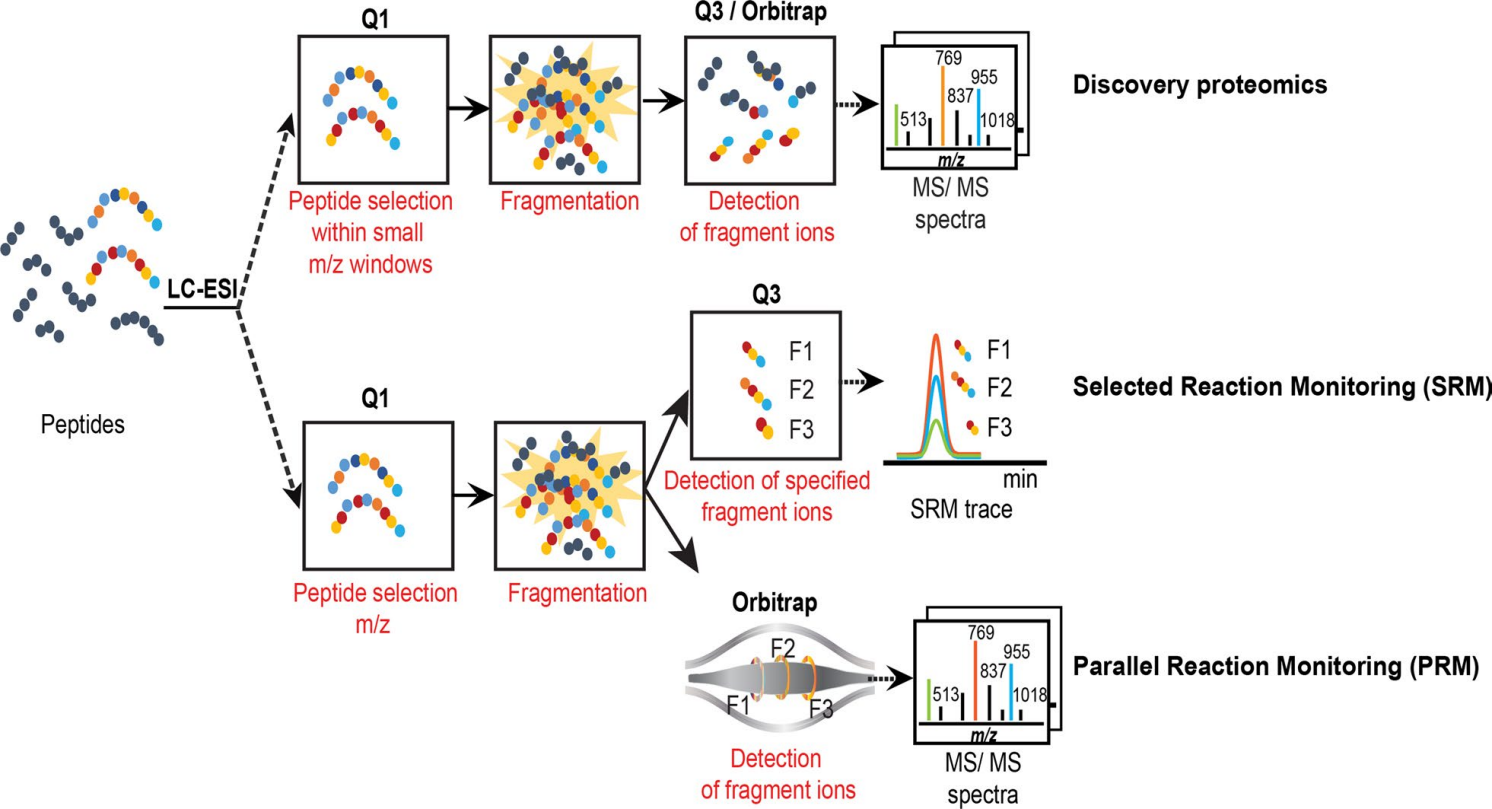
Principles of discovery and targeted proteomics approaches. Major steps of discovery and targeted mass spectrometry analyses are schematized. Discovery proteomics (top panel) characterizes the global composition of a protein sample. With the quadrupole-orbitrap technology, peptide ions within a small window of mass-to-charge (m/z) ratio are isolated in the first quadrupole (Q1) and then fragmented in a collision cell; all ion fragments are finally monitored in the orbitrap analyzer. The processing of resulting MS/MS spectra allows identifying the proteins initially present in the samples (not shown). Targeted proteomics (bottom panel) precisely quantifies a predefined set of proteins. The SRM methodology first selects peptide ions representative of the proteins of interest in the first quadrupole (Q1); they are fragmented in the second quadrupole (Q2); finally, predefined representative ion fragments (F1, F2 and F3) are recorded in the last quadrupole (Q3). The reconstitution of each peptide elution profile, named SRM trace, allows for the integration and quantification of its abundance. The PRM methodology is similar to the SRM pipeline but the last quantification step is not restricted to a predefined set of fragment ions and can consider all of them, recorded in the Orbitrap analyzer

Targeted MS analyses have emerged as an alternative analytical scheme to quantify a predefined set of proteins of interest in a complex protein matrix [28, 29]. The objective of such analyses compares to the use of antibodies against a few proteins of biological interest, yet with the advantages of higher selectivity and straightforward multiplexing. Targeted proteomics by selected reaction monitoring (SRM) consists of selectively recording proteolytic (usually tryptic) peptide sequences that are unique to the proteins of interest. The quantification of such peptides specific of a protein sequence thus informs on the abundance of that protein. SRM originally uses a triple quadrupole mass spectrometer (QQQ), which is able to select, fragment, and quantify the ions corresponding to the peptides of interest (Fig. 1). Briefly, the first quadrupole (Q1) allows selecting the m/z ratio corresponding to a desired peptide. The latter ions then enter a second quadrupole (Q2) in which they get fragmented. Finally, some predefined fragment ions are selected in the third quadrupole (Q3) to be detected. The m/z ratios of the fragments associated with the m/z of the original peptide are called transitions. Recording of the transitions for a given peptide throughout its chromatographic elution peak informs on its abundance in the sample, and by extrapolation, on the amount of the corresponding protein. Isotopically ^13^C/^15^N-labeled synthetic peptides are commonly added to the initial protein sample or proteolytic peptide mixture to ascertain proper recording of the endogenous peptide transitions [30, 31]. Another targeted MS approach, named parallel reaction monitoring (PRM), has been developed more recently. It relies on MS instruments generally used in discovery analyses (e.g., Q-Exactive instruments). Instead of only recording a selection of peptide-fragment transitions, this method allows acquiring on each targeted peptide a complete MS/MS spectrum with high-resolution and mass accuracy on fragment ions [32, 33], which allows better discriminating fragments of the targeted peptides from possible contaminants.

In the context of histone characterization, SRM analyses have been successfully used to investigate their PTMs. Zhang et al. evaluated the level of histone H3 acetylation in human brain tissue with advanced Alzheimer’s disease as compared to neurological controls [34]. Darwanto et al. successfully developed an SRM method to quantify low-abundance histone modifications [35]. This work notably focused on the correlation between H3K120 ubiquitination and H3K79 methylation. Recently, a study proposed a targeted mass spectrometry approach to quantify histones H3 and H2B for a clinical application in patients affected by a critical bacteriaemic septic shock [36]. Finally, PRM was used to monitor modifications on human and mouse H3 and H4 and identified new methylation and acetylation sites [37, 38].

In this report, we developed SRM- and PRM-based methods to quantify a maximum number of H2A and H2B variants in a multiplexed assay. Our goal was to be able to identify and quantify more reliably histone variants from a crude histone extract (around 1500 proteins) than discovery analyses would allow doing. A list of histone variants we recently published was explored to select histone isoforms amenable to a targeted proteomic analysis [8]. Tryptic peptides were identified that are specific for each of the selected histone variants, and extensive LC–MS/MS analysis confirmed that these “signature peptides” bear no PTM or were modified at very low levels. The SRM and PRM methods were successfully implemented and used to analyze the abundance of H2A and H2B histone variants during sperm differentiation in mouse. This choice was motivated by the fact that almost all known histone variants are expressed during spermatogenesis. The implementation of the method confirmed the expression profiles of many testis-specific histones and demonstrated the existence of a predicted H2A.L.1 isoform. Finally, we established by PRM the over-expression pattern of H2A.L.1 isoforms in spermatids of a mouse model of male infertility.

## Results

### Theoretical histone peptides relevant for a targeted proteomic analysis

**Fig. 2:**
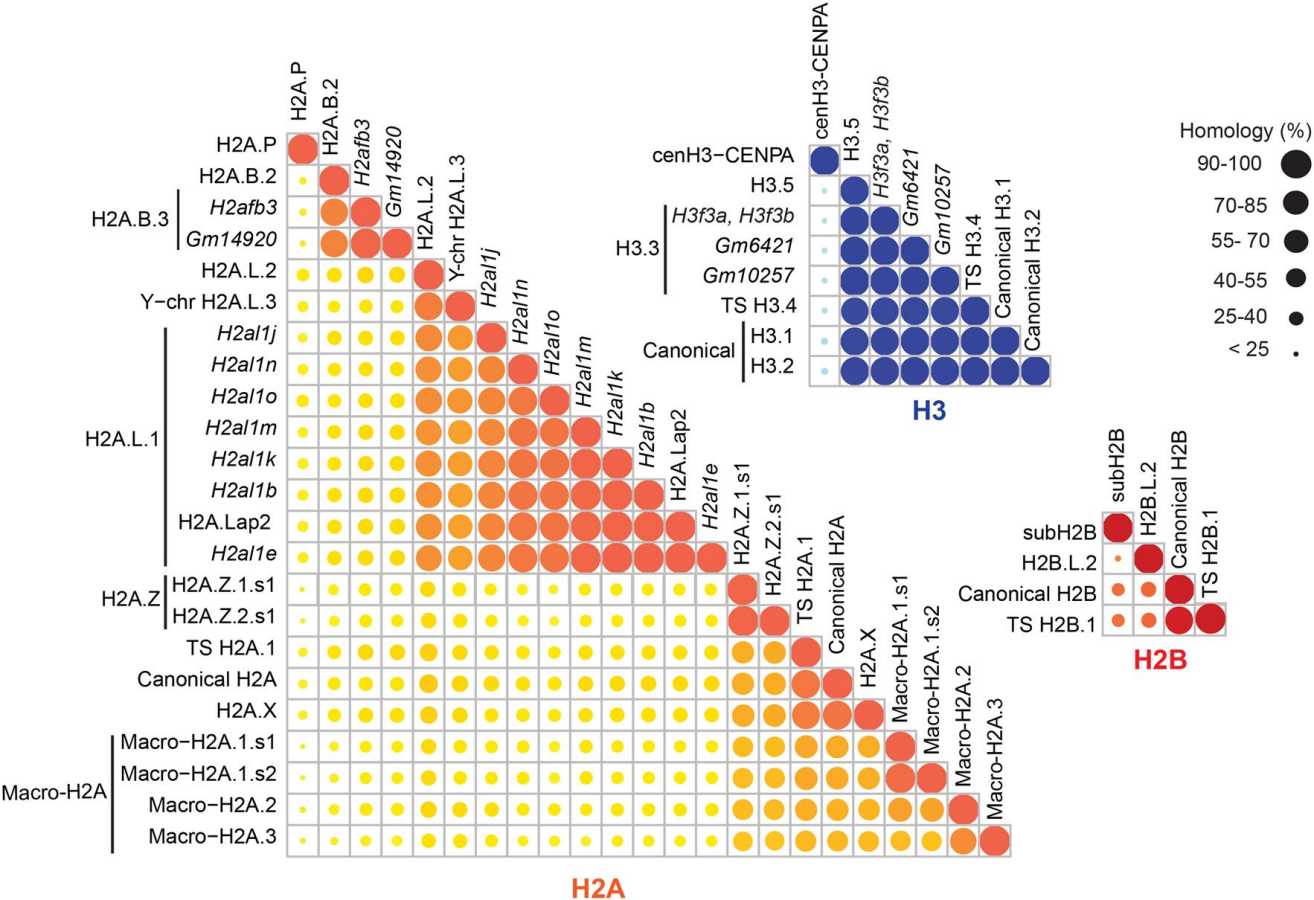
Sequence similarities between H2A, H2B and H3 histone variants. The similarity between H2A, H2B, and H3 variants is displayed in orange, red, and blue, respectively. Sequences were aligned with Clustal Omega tools available on the EMBL-EBI Website [39]. The size of plotted disks is proportional to the percentage of similarity between histone variants

The difficulty to perform a functional analysis of histone variants with traditional biochemical approaches comes from their strong sequence homology that exceeds 90% for many of them (Fig. 2). We hypothesized that targeted proteomics could represent an interesting alternative to the use of antibodies in order to obtain specific detection and precise quantification of histone variants. Such MS analyses require selecting peptides specific of each histone variant that can be successfully analyzed by MS. Since histones are well known to be highly decorated by a multitude of PTMs, finding non-modified (or minimally modified) sequences specific of each variant was a particularly challenging task.

**Fig. 3:**
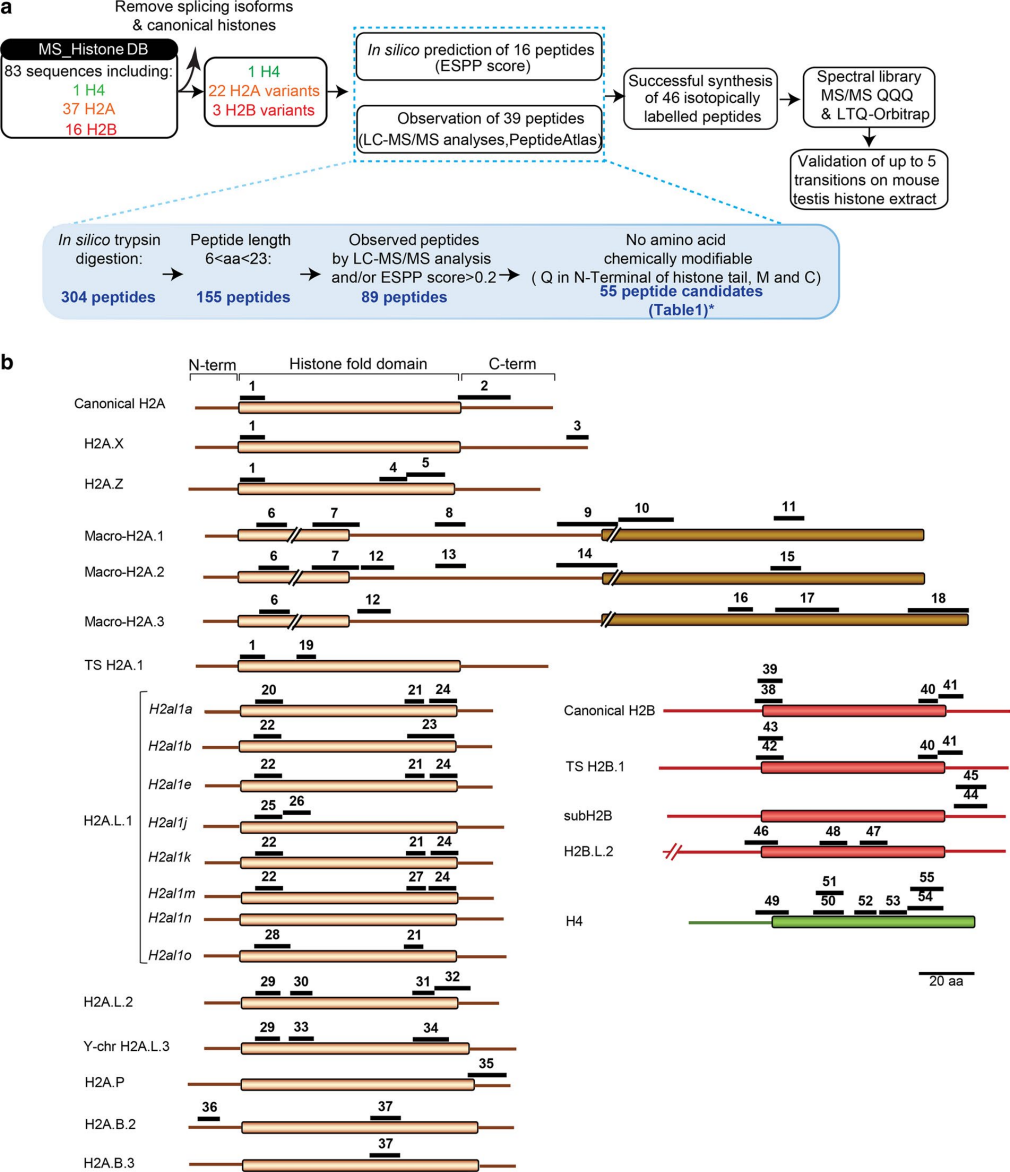
Signature peptides used to quantify H2A and H2B variants by targeted proteomics. **a** Strategy used to select the signature peptides and validate their compatibility with targeted proteomic analysis. The sequences of 22 H2A and 3 H2B variants were obtained from our recently published list of mouse histone variants (MS_histone_DB, [8]). *In silico* digestion of these sequences produced a theoretical list of peptides, which were ranked according to their computed ESPP score, predictive of their compatibility with MS analysis [41]. Fifty-five peptides were selected and further analyzed to monitor the potential presence of post-translational modifications, which could interfere with their analysis by targeted proteomics. This analysis excluded seven of them (see Table 1). Then, heavy standard peptides, ^13^C,^15^N-labeled, were synthesized and analyzed on different MS instruments (LTQ-Orbitrap Velos, QTRAP 5500) to acquire full MS/MS spectra and create spectral libraries. They were used to select up to five more intense SRM transitions for each peptide. **b** Selected signature peptides presented on their corresponding histone variants. They are presented as black bars and numbered according to Table 1. Histone fold domains, also called globular domains, are presented as a rectangle for each histone, surrounded by N- and C-terminal tails. H2A (orange), H2B (red), H4 (green)

We referred to MS_HistoneDB, a comprehensive and non-redundant list of 83 mouse histone variants recently published by our group to determine the protein sequences of interest [8]. We chose to digest protein samples with trypsin, the most classically used protease in proteomics: Trypsin indeed provides highly reproducible proteolysis and generates peptides ending with the basic Lys/Arg residues which are favorable for ionization and MS/MS fragmentation [40]. The in silico digestion of the 83 mouse histone sequences with trypsin produced a list of 304 theoretical peptides. From this list we selected peptides more likely to be successfully identified by MS by considering the following criteria (Fig. 3a). First, peptide length had to be comprised between 6 and 23 amino acids, which reduced the list to 155 peptides. The fact that histones are rich in K/R residues, leading to very small peptides in some protein regions, accounted for this significant peptide list reduction. Second, their detectability potential using MS was estimated using the ESP Prediction tool [41] that calculates an ESPP score for each peptide sequence. Based on published recommendations and our LC–MS/MS discovery analyses of histones from mouse testes, a score threshold was set to 0.2 [41]. This led to 89 remaining peptides. Finally, by using the software Skyline fed with MS_HistoneDB, each peptide was confirmed to be unique for its histone entry among the mouse proteome, or shared by a small group of histone variants/isoforms [28, 29]. The list of peptides preselected for targeted MS analysis was compared to experimental data available in public databases, notably PeptideAtlas [42]. Next, the presence of modifications on the signature peptides was carefully examined, because a significant stoichiometry would bias the quantification of the histone variants which the peptides represent. Chemical modifications possibly occurring in vitro were also considered: sample preparation (in-gel digestion) and LC–MS/MS analysis classically use low pH solutions. Peptides containing methionine residues, prone to oxidation, or with an N-terminal glutamine, readily converted into pyroglutamate under acidic conditions, were then excluded when possible, in agreement with formerly published guidelines on SRM assay development [43].

The six H3 variants have nearly identical sequences with very minor variations. Selecting specific tryptic peptides that met the above-listed criteria was therefore not feasible (Additional file 1) [28]. Indeed, five peptides specific of a single variant were obtained by in silico digestion, one and four covering the canonical histone H3.1 and cenH3, respectively. However, two peptides may be subject to a pyroglutamylation in acidic conditions (Additional file 1, peptides A and B), while three others are preceded by several R/K neighboring residues, which renders trypsin cleavage site uncertain (Additional file 1, peptides C, D and E, see further comments on this issue below); peptides D and E also contain more than 30 amino acids (Additional file 1). We then decided to focus our targeted analyses on H2A and H2B variants.

Among the 55 theoretical peptides allowing the analysis of 22 H2A, 3 H2B variants and histone H4, 31 peptides met all the above-listed requirements for SRM analyses (Additional file 2). They could notably differentiate between highly similar isoforms of histone variants. Peptides P20 and P22 thus differ by one amino acid only, while peptides P20 and P25 exhibit an inversion of two amino acids and can discriminate between H2A.L.1 isoforms (Fig. 3b and Table 1). Such selectivity can be achieved by peptide-based targeted proteomics and is very unlikely to be apprehended by traditional biochemical approaches, nor by proteomics top-down or middle-down analyses.

**Table 1:** H2A, H2B and H4 signature peptides

Peptide N°	Protein	Peptide sequence	ESPP score	Heavy peptide identified	Light peptide identified	Identified in our discovery analyses	Recorded in PeptideAtlas
1	Canonical H2A, H2A.J, H2A.X, H2AZ.1, H2AZ.2, TS H2A.1	AGLQFPVGR	0.739	Yes	Yes	Yes	PAp00032266
2	Canonical H2A, H2A.J, TS H2A.1	VTIAQGGVLPNIQAVLLPK^a^	0.224		Yes	Yes	PAp00008634
3	H2A.X	ASQASQEY	0.278	No	No	Yes	PAp00852754
4	H2AZ.1, H2AZ.2	GDEELDSLIK	0.519	Yes	Yes	Yes	PAp00067999
5	H2AZ.1, H2AZ.2	ATIAGGGVIPHIHK	0.522	Yes	Yes	Yes	PAp00032640
6	Macro-H2A.1, Macro-H2A.2, Macro-H2A.3	AGVIFPVGR	0.575	Yes	Yes	Yes	PAp00413759
7	Macro-H2A.1, Macro-H2A.2	HILLAVANDEELNQLLK	0.222	Yes	Yes	Yes	PAp00069466
8	Macro-H2A.1	LEAIITPPPAK	0.713	Yes	Yes	Yes	PAp00429995
9	Macro-H2A.1	AASADSTTEGTPTDGFTVLSTK	0.517	Yes	Yes	No	PAp00380945
10	Macro-H2A.1	NGPLEVAGAAISAGHGLPAK	0.723	Yes	Yes	Yes	PAp00389241
11	Macro-H2A.1	SIAFPSIGSGR	0.796	Yes	Yes	Yes	PAp00077504
12	Macro-H2A.2, Macro-H2A.3	GVTIASGGVLPR	0.866	Yes	Yes	Yes	PAp00519811
13	Macro-H2A.2	SETILSPPPEK	0.580	Yes	No	Yes	
14	Macro-H2A.2	EGTSNSTSEDGPGDGFTILSSK	0.499	No	No	Yes	
15	Macro-H2A.2	SVAFPPFPSGR	0.578	Yes	Yes	Yes	
16	Macro-H2A.3	NCLSAAEIR	0.726	Yes	No	No	
17	Macro-H2A.3	SPVAETASPGRPGDPQGHLGSLR	0.664	Yes	No	No	
18	Macro-H2A.3	AGDGQTGHQVALSGSGGEGGSA	0.504	No	No	No	
19	TS H2A.1	QGNYAQR	0.089	Yes	No	No	
20	H2A.L.1-*H2al1a*	GELPFSLVDR	0.821	Yes	Yes	Yes	
21	H2A.L.1-*H2al1a*, *H2al1e*, *H2al1k*, *H2al1o*	IAPEDVR	0.299	No	No		
22	H2A.L.1-*H2al1b*, *H2al1e*, *H2al1k*, *H2al1m*, *H2al1n*	GELPLSLVDR	0.814	Yes	Yes	Yes	
23	H2A.L.1-*H2al1b*	IAPEDVHLVVQNNEQLR	0.301	Yes	No	Yes	
24	H2A.L.1-*H2al1a*, *H2al1e*, *H2al1k*, *H2al1m*	LVVQNNEQLR	0.596	Yes	Yes	Yes	
25	H2A.L.1-*H2al1j*	GEFPLSLVDR	0.821	Yes	No		
26	H2A.L.1-*H2al1j*	FLPEGNHSGR	0.445	Yes	No		
27	H2A.L.1-*H2al1m*	VTPEDVR	0.216	Yes	No		
28	H2A.L.1-*H2al1o*	GELPLSLVDHFLR	0.262	Yes	No		
29	H2A.L.2, Y-Chr H2A.L.3	AELQFPVSR	0.844	Yes	Yes	Yes	
30	H2A.L.2	FLREGNYSR	0.289	No	No		
31	H2A.L.2	IAPEHVCR	0.438	Yes	No	Yes	
32	H2A.L.2	VVQNNEQLHQLFK^a^	0.322		Yes	Yes	
33	Y-Chr H2A.L.3	FLGEGIYSR	0.433	Yes	No		
34	Y-Chr H2A.L.3	IAPEHVCQVVQNK	0.464	Yes	No		
35	H2A.P	NAPFSLFDEMPGPR	0.673	Yes	No		
36	H2A.B.2	NTENCLQR	0.261	Yes	No		
37	H2A.B.2, H2A.B.3	LLELAGNEAQR	0.777	Yes	No		
38	Canonical H2B	KESYSVYVYK	0.255	Yes	Yes	Yes	PAp00035223
39	Canonical H2B	ESYSVYVYK	0.359	Yes	Yes	Yes	PAp00066832
40	Canonical H2B, TS H2B.1	EIQTAVR	0.331	Yes	Yes	Yes	PAp00066243
41	Canonical H2B, TS H2B.1	LLLPGELAK	0.517	Yes	Yes	Yes	PAp00073476
42	TS H2B.1	KESYSIYIYK	0.227	Yes	Yes	Yes	PAp00379050
43	TS H2B.1	ESYSIYIYK	0.311	Yes	Yes	Yes	
44	subH2B	KLATLAVTFGSK	0.608	Yes	Yes	Yes	
45	subH2B	LATLAVTFGSK	0.571	Yes	Yes	Yes	
46	H2B.L.2	NSFAIYFPK	0.398	Yes	Yes	Yes	
47	H2B.L.2	SVNILDSFVK	0.421	Yes	Yes	Yes	
48	H2B.L.2	IASEASFLAR	0.728	Yes	Yes	Yes	
49	H4	DNIQGITKPAIR^a^	0.845		Yes	Yes	PAp00033165
50	H4	RISGLIYEETR^a^	0.548		Yes	Yes	PAp00006681
51	H4	ISGLIYEETR^a^	0.667		Yes	Yes	PAp00035058
52	H4	VFLENVIR^a^	0.259		Yes	Yes	PAp00038639
53	H4	DAVTYTEHAK^a^	0.333		Yes	Yes	PAp00039819
54	H4	KTVTAMDVVYALK^a^	0.505		Yes	Yes	PAp00072198
55	H4	TVTAMDVVYALK^a^	0.402	_	Yes	Yes	PAp00008048

The carbamidomethylated or oxidized forms of peptides containing cysteine or methionine residues, respectively, were also monitored. “Yes” indicates that peptides were successfully detected by LC-SRM or LC–MS/MS analysis and “No” indicates they failed to be

^a^Peptides monitored only in their endogenous form

As no H4 variant has been described in mouse, this histone is particularly adapted for inter-sample normalization. Peptide ISALVYEETR from yeast histone H4 corresponding to the sequence ISGLIYEETR (P51) in mouse has already been successfully used to normalize total histone abundance by SRM [44]. Since we also identified the latter sequence with a missed cleavage by LC–MS/MS analysis, we additionally considered the sequence RISGLIYEETR (P50). Finally, a third peptide, P52, was also recorded to quantify H4 abundance (Table 1, Fig. 3). Peptides P53–P55 were originally further included in the SRM methodology to start with a large enough set of candidate peptides, but were finally not considered for H4 quantification, due to weak signal or excessive variability of quantitative measurements for P53–55.

### Trypsin missed cleavages in signature peptides

**Fig. 4:**
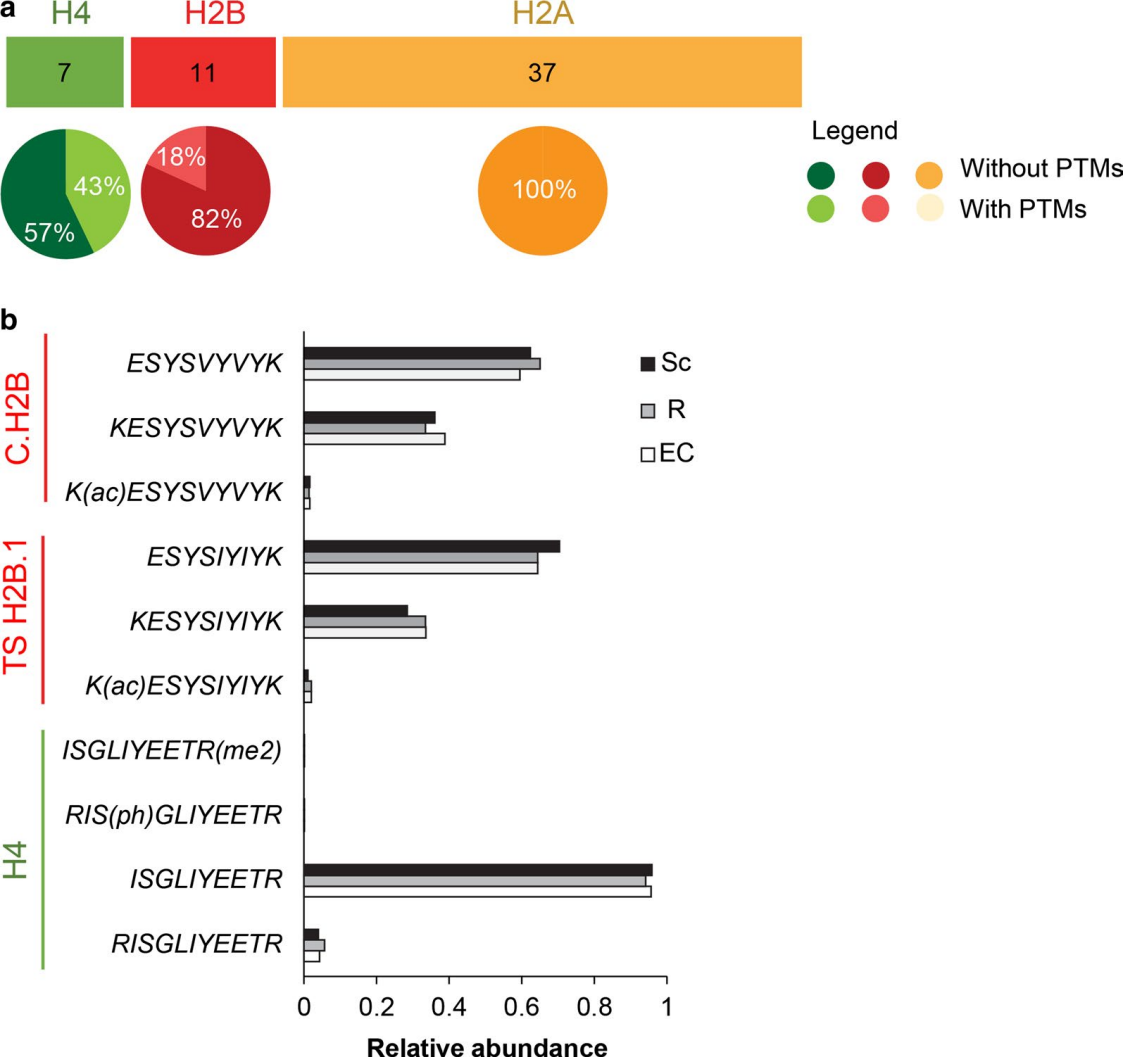
Signature peptides are mostly devoid of post-translational modifications. **a** Numbers of H2A, H2B and H4 peptides used in the targeted proteomic analysis that were identified to be fully non-modified or modified to some extent by discovery LC–MS/MS analyses. **b** Abundance of modified versus non-modified forms of the signature peptides of H2B, TS H2B.1 and H4. Analyses were performed on spermatocytes (Sc), round spermatids (R), elongating and condensing spermatids (EC)

The list of 55 selected peptides contains five pairs of sequences differing by one missed cleavage, such as (K)ESYSIYIYK for TS H2B.1 (peptides P38–39), and also the peptide pairs P42–43, P44–45, P50–51, and P54–55. For histone H4, we monitored P50–52 to normalize the amounts of H2A and H2B histone variants, as described above. We observed that quite stable relative proportions of the fully and non-fully cleaved peptide versions were produced across histone samples obtained by classical acid-based extraction from spermatocytes, round spermatids and elongating/condensing spermatids when the protein samples were digested in parallel (Fig. 4). The case of RISGLIYEETR (P50) and ISGLIYEETR (P51) for H4 is shown in Additional file 3. Besides, we observed that 21 out of the 55 selected peptides had further neighboring Lys/Arg residues on the N- or C-terminal side which could lead to peptides with one or several missed cleavages. Peptide ASQASQEY from H2A.X is thus preceded by two Lys residues. Such peptides are usually excluded from targeted proteomic analyses because the fractions of peptides with and without non-cleaved K/R residues may vary between independent sample preparations, which would impact protein quantification. However, the limited number of options to quantify some histone variants (e.g., H2A.X, H2A.L.1, and H2A.B) forced us to consider such peptides. The variably cleaved forms were systematically monitored by targeted analysis, and barely any or no signal was measurable for the peptides with missed cleavages, probably because we always performed our in-gel digestion protocol with an excess of protease.

To conclude, a list of 55 theoretical signature peptides was established to detect and quantify 100% of H2A and H2B histone variants. It also included peptides of H4 to be used for normalization. Most of them (71%, 39 peptides) were experimentally detected while 29% (16 peptides) were only predicted by an in silico analysis (Table 1).

### Signature peptides are devoid of post-translational modifications

Histones are proteins particularly challenging to follow by SRM to get protein abundances, since they are decorated with a wealth of dynamic PTMs [45]. Histones have two structurally distinct features: a globular domain that is responsible for the formation of core nucleosomal particles and unstructured tails protruding from this core particle. The histone globular domains play an important structural role in the assembly of nucleosomes and bear fewer modifications than the N- or C-terminal tails [46]. For this reason, signature peptides were preferentially chosen within the histone globular domains (Fig. 3b). Nonetheless, the absence of PTMs at significant stoichiometry on the signature peptides was experimentally confirmed, by discovery analysis of gel-separated histone samples. Mouse testis tissue was chosen as a source of histones to be analyzed. It showed the advantage of providing abundant quantities of histones at different stages of spermatogenesis, which facilitated the development of this methodology. In addition, different publications have documented the presence of many histone variants in this tissue, including many testis-specific ones [14]. Finally, many histone modifications have been described during spermatogenesis [47, 48]. Using such a tissue to confirm that PTMs are not significantly present on the selected signature peptides, thus strongly supports the relevance of these peptides in other biological contexts. Histones were purified from different stages of sperm differentiation, with meiotic spermatocytes and post-meiotic round and elongating/condensing spermatids. PTMs (acetylation, mono- and dimethylations, phosphorylations) were extensively searched in the signature peptides by discovery LC–MS/MS analysis of histone samples extracted from testis and migrated on a gel. Modifications were identified on 5 out of the 55 peptides listed in Table 1 (Fig. 4a). The modified forms were quantified by integrating the area under the chromatographic peaks of the modified and non-modified peptide versions within the LC–MS/MS analyses. They never represented more than 2% of the total quantity of the peptide detected (Fig. 4b). We then concluded that the presence of PTMs on the signature peptides was unlikely to interfere significantly with the quantification of the corresponding proteins.

### Development of an SRM assay to quantify histone variants

The 55 peptides of interest were synthesized in a heavy-isotope-labeled version with incorporation of ^13^C/^15^N atoms into the C-terminal Arg or Lys residues. We later call them “standards.” Unfortunately, P2 and P32 could not be synthesized but the natural sequences were still recorded in the follow-up analyses. The heavy peptides were mixed and analyzed by discovery analysis on a Qtrap instrument. Whole fragmentation spectra were acquired, from which higher-intensity fragment ions were selected to establish peptide–fragment transitions [28]. For each peptide of interest, 3–5 highly responding fragment ion candidates were selected [28]. Most histone variants were represented by a single peptide, and the analyzed samples were relatively complex with ~ 1000 to 1500 proteins identified per exploratory LC–MS/MS analysis. Selecting 3–5 transitions per peptide limited the risk of quantification failing due to the contamination by another peptide of close m/z ratio that would produce overlapping transitions. Of note, 25 signature peptides of H2A variants contain one or several proline residues that are detrimental to fragmentation in continuous y-type ion series. Indeed, y-type ions ending with the Pro residue(s) are highly stabilized during MS/MS fragmentation. In such cases, it happened that only fewer than five transitions could be selected. Yet the Pro-ending y-type ions of characteristic higher intensity constituted excellent transitions.

The detectability and signal specificity of endogenous peptides was next tested by spiking the mixture of labeled peptides in a tryptic digest of histones extracted from mouse testes. The perfect co-elution of endogenous peptides with their synthetic counterparts and the similarity of the fragmentation patterns were confirmed.

Finally, 550 transitions were monitored by LC-SRM analysis (Additional file 4). Forty-one out of 46 standard peptides had symmetrical and narrow chromatographic elution profiles, with intensities at least five times higher than the background signal, while P3, P14, P18, P21, and P30 were not successfully identified by LC-SRM analysis, probably due to weak ionization efficiency. Despite a high amount of P19 standard (for TS H2A.1) spiked in the sample (estimated to be at about 119 pmol/μL, see Additional file 5), the intensity of the SRM trace remained weak. The possible pyroglutamylation in the N-terminal glutamine of the endogenous peptide sequence and its weak ESPP score could explain this result (Table 1). Of note, four of the five poorly detected peptides above were not recorded in PeptideAtlas.

### Performance of the SRM assay

**Fig. 5:**
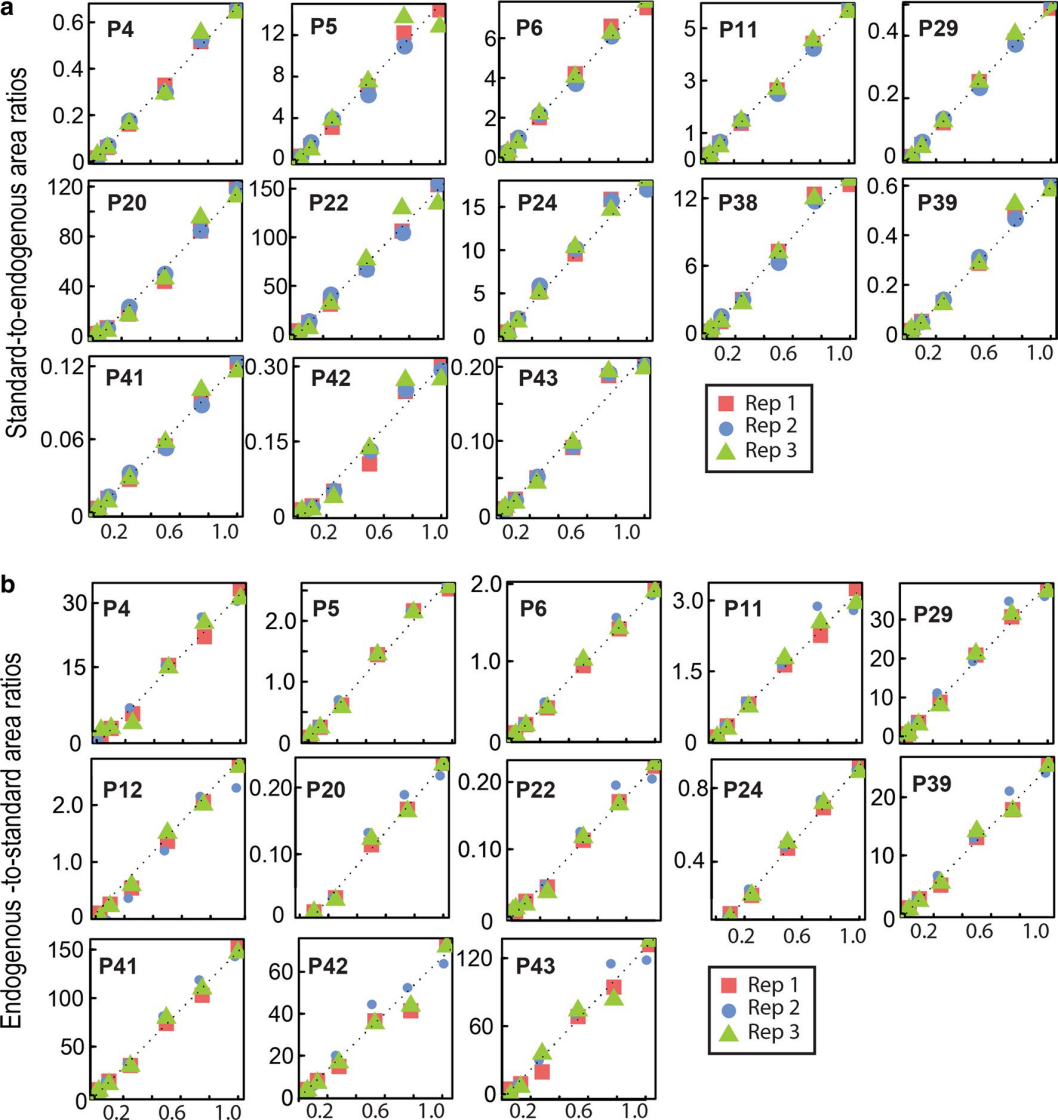
Evaluation of the linearity of SRM quantification. **a** The SRM quantification is linear, using an increasing quantity of each isotopically labeled signature peptide spiked in a constant amount of protein matrix (acid-extracted histones from mouse testis). Please refer to the “Methods” section for experimental details. Data were normalized as described in Ref. [49]. **b** Similar results were obtained when a constant quantity of isotopically labeled signature peptide was spiked in an increasing amount of protein matrix (acid-extracted histones from mouse testis)

The analytical performances of the SRM method were evaluated by stable isotope dilution (SID) over a 100-fold dilution range [31]. For this purpose, increasing quantities of heavy standard peptides were added in a constant quantity of testis histone extract. Figure 5a shows representative response curves of signature peptides, in which standard-to-endogenous ratios are plotted against their theoretical dilution factor. Excellent linear responses were observed for all peptides (*R*^2^ > 0.96, Fig. 5a, Additional file 6). The median CV for technical replicates was under 30% for 75% of the peptides, which is correct when the objective is to perform relative quantification.

The former dilution series was well suited to test whether the method allowed detecting abundance variations of a few histone variants in a globally constant complex sample. However, samples can be highly variable when studying a biological system. This is the case for histones extracted from cells at different stages of spermatogenesis. In this example, the variability is due to the chromatin dynamics throughout sperm differentiation, in particular, the progressive replacement of histones by transition proteins and protamines during this process (for review see [50, 51]). In the present context of study and in others consisting of comparing significantly different histone samples, it was necessary to also test the response linearity by varying the amount of endogenous material and following the corresponding peptide signals while keeping the standard peptides constant. Then, a constant quantity of standard peptide was added in an increasing amount of histone testis extract (Fig. 5b). The monitored abundances correlated very well with the dilution factors (*R*^2^ > 0.96), even though the highly variable matrix could have been expected to significantly impact the ionization efficiency of the peptides of interest (Additional file  6).

### Quantifying histone variants during mouse spermatogenesis

**Fig. 6:**
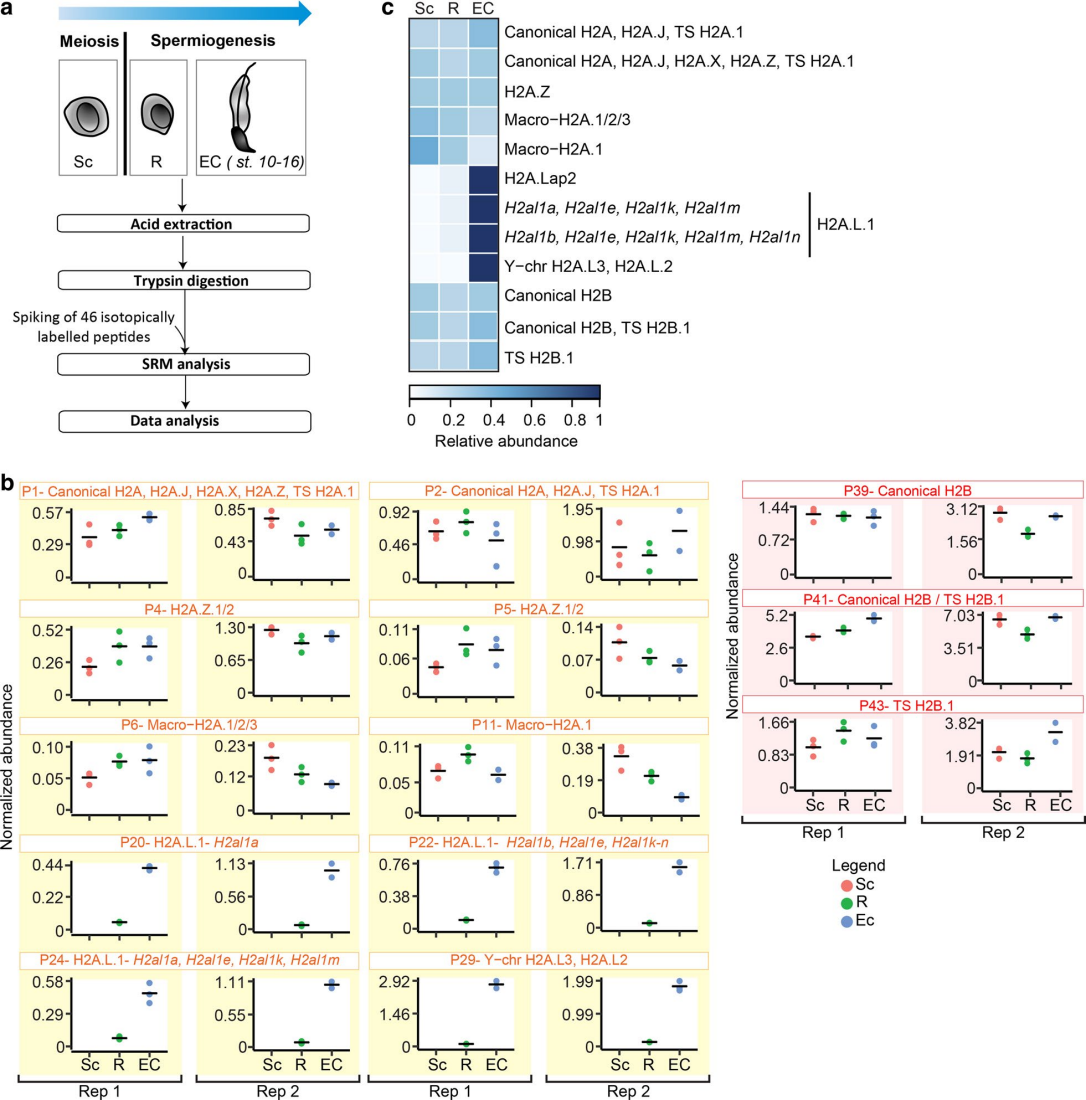
SRM-based quantification of H2A and H2B variants during mouse spermatogenesis **a** Experimental design. Spermatogenic cell fractions were analyzed [meiotic spermatocytes (Sc); round spermatids (R); elongating and condensing spermatids (EC)]. Histones were extracted with sulfuric acid, digested by trypsin and analyzed by LC-SRM. **b** LC-SRM quantification of the abundance of histone variants during spermatogenesis. Data were normalized to H4 levels as described in the “Methods” section and in Additional file 3. Two independent biological replicates are presented for each protein (replicate 1 and replicate 2) and were analyzed in technical triplicates. **c** Heat maps representing the abundance of H2A and H2B variants during spermatogenesis

The validated SRM methodology was implemented to investigate the abundance of H2A and H2B variants during mouse spermatogenesis (Fig. 6a).

*H2A histone variants* The two isoforms of H2A.Z, H2A.Z.1, and H2A.Z.2, are highly similar and differ by only three amino acids [52]. Two tryptic peptides shared by both H2A.Z isoforms could be quantified during spermatogenesis. When considering the average of signals detected on P4 and P5, we found that the amount of H2A.Z was globally constant through spermatogenesis (Fig. 6). Greaves et al. assessed the relative amount of this variant during spermatogenesis by indirect fluorescence generated by affinity-purified H2A.Z antibodies [53]. They observed a moderate increase in H2A.Z protein (by a factor of about 1.5) in round spermatids compared to pachytene spermatocytes. Such an abundance ratio is often not distinguishable from 1 in proteomics measurements.

Two macro-H2A genes were identified in mammals, *H2AFY* and *H2AFY2*, that encode macro-H2A.1 and macro-H2A.2 isoforms, respectively. MacroH2A is largely expressed in mouse testis [54] and mainly associated with transcriptionally inactive domains [55, 56]. MacroH2A.1 is more expressed during the early pachytene stage of spermatogenesis, where it is associated with sex vesicles containing sequestered X- and Y-chromosomes [57]. Even though we did not analyze histones from this stage, targeted analysis of peptide P11 specific of Macro-H2A.1 showed decrease by a factor of 2 toward the end of spermatogenesis [57]. Finally, the abundance of peptide P6, shared by Macro-H2A.1/2/3, seemed to be constant through spermatogenesis (Fig. 6).

H2A.X would have been quantifiable by one peptide only, namely P3 (Table 1). Yet this peptide could not be detected, probably due to poor ionization efficiency, as predicted from its low ESPP score (0.278).

H2A.L.1 variant, also called H2A.Lap.2, is encoded by the genes *H2al1a*, *H2al1c*, *H2al1d*, *H2al1f*, *H2al1g*, *H2al1h*, and *H2al1i*. The sequence of H2A.L.1 and H2A.L.2 are very close, with 72% of sequence identity. This explains why, to the best of our knowledge, no H2A.L.1- or H2A.L.2-specific antibody has yet been described [14, 58, 59]. Both H2A.L.1 and H2A.L.2 variants are strongly enriched in elongating and condensing spermatids, and a recent study demonstrated that H2A.L.2 variant participates in the final spermatic chromatin organization [58]. The direct contribution of H2A.L.1 is still unclear and which H2A.L.1 isoforms are expressed at the protein level remains to be characterized. In the present study, peptide P20, specific of the H2A.L.1 isoform encoded by *H2al1a* gene, was quantified by SRM and its abundance through the three spermatogenesis stages appeared in complete agreement with its published expression profile [14, 56]. Seven other putative H2A.L.1 isoforms, that would differ by a few amino acids, have been inferred by homology or only identified at the transcript level [8]. Peptide P22 is shared by the H2A.L.1 isoforms encoded by *H2al1b*, *H2al1e*, *H2al1k*, *H2al1m,* and *H2al1n* genes (see P20 vs. P22 peptides indicated in Table 1). Interestingly, its detection by MS confirmed the existence of the protein product of at least one of these genes. Thus, it accumulates in the last stage of sperm differentiation, similarly to the *H2al1a*-encoded protein (Fig. 6).

*H2B histone variants.* In mice, TS H2B.1 is highly expressed in testis and participates in the establishment of a sperm-specific chromatin structure [14, 58, 60]. Its abundance was estimated with peptides P42 and P43 and was observed to be constant over the three stages of spermatogenesis analyzed. Similarly to previous studies, although present in whole testis extracts, H2B.L.2 was not detected in germ cells [14]. This result correlates with the fact that H2B.L.2 mRNA was detected at a very low level in meiotic, as well as post-meiotic, germ cells [14].

Altogether, our SRM methodology confirmed that about 70% of H2A and H2B variants can be quantified in a single multiplexed assay, with results confirming abundance profiles previously published using antibodies [14, 58–60]. The most prominent discovery brought by our analyses is the identification, for the first time at the protein level, of a new isoform of H2A.L.1. This isoform dramatically increases in abundance in the course of spermatogenesis, mirroring the variations of the originally studied variant H2A.L.1. This information could only be obtained by the discriminative power of proteomics that can distinguish sequences differing by one single residue.

### Quantification of histone variants in a mouse model of male infertility

The established methodology was then tested on a mouse model of male infertility. The transcription regulator SLY is encoded by the Y-chromosome and is expressed only in spermatids where it controls the expression of hundreds of sex chromosome-encoded genes, including several histone variants [61, 62]. This gene was knocked down by a transgenic approach of shRNA in the mouse (Sly-KD males) resulting in defects in sperm differentiation with abnormal chromatin compaction and increased sperm DNA damage [63, 64]. At the transcript level, many X- and Y-chromosome-encoded genes are upregulated in Sly-KD round spermatids; among them are the spermatid-specific genes *H2afb3* and *H2al1*, which encode histone variants H2A.B.3 and H2A.L.1, respectively. Autosomal genes encoding H2A variants (such as *H2al2*) were not found deregulated [64]. It was not possible to confirm upregulation of H2A.L.1 at the protein level because anti-H2A.L.2 antibody cross-reacts with H2A.L.2. We therefore sought to apply our methodology to quantify histone variants in Sly-KD round spermatids. For this application, the SRM methodology was adapted to a PRM approach to gain sensitivity (Additional file 7). Using PRM also improved the detection of peptide P23, specific for the H2A.L.1 isoform encoded by *H2al1b*, which was hardly detectable by LC-SRM.

**Fig. 7:**
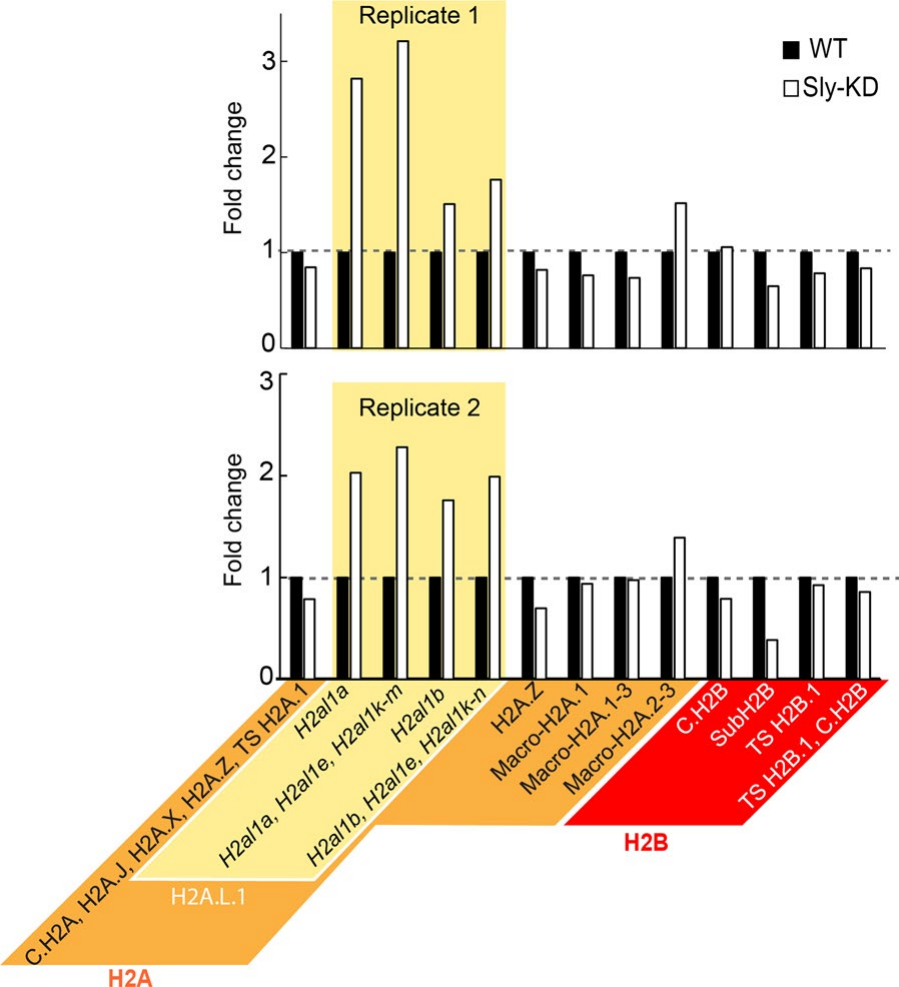
Quantification of the abundance of H2A.L.1 isoforms in Sly-KD mice. The relative abundance of H2A and H2B variants was quantified by PRM in round spermatids from WT and Sly-KD mice. Two independent biological replicates are presented (top and bottom panels). H2A.L.2 and Y-ChrH2A.L.2 are expected to be expressed at similar levels between Sly-KD and WT spermatids [64] and peptide P29, shared by both variants, was used to normalize the abundance of peptides specific to H2A.L and subH2B (H2B.L.1) variants. The abundance of the other histone variants was normalized to H4 (P50–52). For more details, please refer to the “Methods” section

PRM analyses also identified another H2A.L.1 isoform and showed that all H2A.L.1 isoforms are indeed over-expressed in Sly-KD compared to WT round spermatids (Fig. 7) [64]. Discovery LC–MS/MS analyses confirmed that no PTM was detectable on the signature peptides used for this quantification, so that peptide abundances truly reflected histone variant abundances (Additional file 8). Even though H2A.B.3 is upregulated at the mRNA level in Sly-KD round spermatids [64], its specific peptide P37 could not be quantified by PRM. As the standard peptide ionized well and could be detected by SRM, the very low abundance of its endogenous counterpart probably explains its non-detection).

## Discussion

In this study, we developed a targeted proteomics approach to quantify a maximum of histone variants in a single assay. In-depth analysis of the sequences of all mouse histone variants defined a set of signature peptides for 100% of H2A and H2B histone variants. They can be produced using trypsin and standard proteomics sample preparation. These peptides were experimentally validated: They could be detected on standard mass spectrometers and are mostly devoid of PTMs. They were used within an SRM methodology to quantify histone variants during sperm differentiation, demonstrating for the first time the dramatic abundant increase in the protein paralogs of H2A.L.1 encoded by genes *H2al1a* and by genes *H2al1b*, *H2al1e*, *H2al1k*, *H2al1m,* and *H2al1n* in condensing/elongating spermatids. It is worth adding that our targeted proteomic approaches led to the identification of a yet undescribed H2A.L.1 isoform (encoded by *H2al1b*). Furthermore, the SRM methodology was transposed to the PRM technology, which successfully showed that testis-specific histone variants, including H2A.L.1 isoforms, are deregulated in a mouse model of male infertility. This observation is important for the pathophysiology of the SLY-KD mouse model: The increased amount of H2A.L.1 in spermatids could indeed contribute to the sperm chromatin remodeling defects and associated male infertility observed in these males [64]. H2A.L.1 is highly similar to H2A.L.2 in terms of sequence and pattern of expression. Since H2A.L.2 knockout has been shown to lead to defective sperm chromatin reorganization [58], the respective contribution of H2A.L.1 isoforms and H2A.L.2 remains to be studied.

The sequence similarity between histone sequences makes it challenging to differentiate canonical histones and their variants at the protein level via antibody-based techniques. The development of MS methods now allows researchers to specifically characterize histones and their PTMs using traditional discovery proteomics. Few studies were interested in providing an analytical tool to monitor simultaneously histone variants with high specificity. A top-down approach was developed to investigate histone variants, although this strategy presents multiple drawbacks including weak sensitivity, difficulties in data interpretation and thus quantification, compared to other MS strategies [65]. Moreover, a top-down approach will likely fail to distinguish two histone variants differing by minor sequence variation from a same variant harboring different PTM combinations. Given that limitation, we decided to develop for the first time a targeted proteomics method enabling to quantify in a single analysis multiple histone variants. We applied the method to investigate variants in the course of spermatogenesis, where many histone variants are expressed dynamically.

The choice of peptides to be followed by SRM requires several considerations including uniqueness, size, detectability, the absence of PTMs or missed cleavage sites [28, 29]. We recently developed two exhaustive and non-redundant protein databases, named MS_HistoneDB, as resources for the proteomic study of mouse and human histones [8]. This resource has been used to identify in silico the peptides which are likely detectable by mass spectrometry and present an interest for the quantification of histone variants by targeted proteomics. However, when studying histones, it is complicated to comply with all the rules established for targeted analyses, because these proteins are enriched in lysine and arginine residues and thus subject to a wealth of PTMs and to trypsin missed cleavages. Despite this challenge, we were able to select 55 peptides (Fig. 3). Transitions were selected to identify the best quantifiable ions to evaluate the abundance of histone variants. The validation of the selected transitions was aided by the addition of isotopically labeled peptides.

The SRM methodology was applied to a tryptic digest of histones extracted from mouse testis using up to six signature peptides per protein or small group of isoforms. The method was effective in detecting five H2A variants in addition to H2A.L.1 isoforms and all H2B variants in the whole testis despite the complexity of the sample, estimated to be around 1000–1500 proteins by discovery analysis. Compared to traditional western blot analysis, the developed SRM assay is extremely time-effective, as it allows in a 1-h analysis to follow several H2A and H2B variants while simultaneously following H4 for normalization.

The developed SRM assay was converted into a PRM assay. This acquisition mode is more time-effective because it does not require selecting transitions to be followed, but records a whole MS/MS spectrum. Moreover, the use of an MS instrument from the latest generation (Q-Exactive) provided better sensitivity that allowed identification of a variant not detected by SRM on a Qtrap instrument, namely H2A.L.1 (encoded by gene *H2al1b*). The differences between PRM and SRM have been recently investigated, using model isotope-labeled peptides and tryptic digests of yeast proteins [32]. Both methods exhibited the same linearity, but PRM performed better with a higher resolution and selectivity on the peptides of interest [66, 67].

Trypsin has been shown to be the most effective protease compared to others [68]. The use of another enzyme such as Glu-C or ArgC, of comparable specificity level and digestion efficacy, would likely help cover additional specific peptides and thus more H2A and H2B histones, particularly H2A.X and testis-specific variants TS H2A.1 and H2A.B. It may also allow addressing the case of H3 variants. Finally, a newer-generation instrument with increased sensitivity would probably allow detecting in lower amounts of specific germ cells additional variants such as H2A.B.3 and H2B.L.2 to assess their abundance variation in the course of spermatogenesis.

## Conclusions

The developed assay is a valuable analytical method to monitor 22 H2A and 3 H2B variants. Our method enabled the comparative quantification of histone variants in spermatocytes, round spermatids and elongating/condensing spermatids. Because histones have been very well conserved from mouse to human, this method can be easily transposed to target human histone variants in a range of applications, including cancers, in which several variants have already been found to be deregulated [69–71].

## Methods

### Analysis of histone variant sequences

The sequences of mouse histones and their variants were obtained from our recently published MS_histoneDB [8]. Putative variants and splicing variants were excluded from the analysis to limit its complexity, and 22 H2A, 3 H2B, and 6 H3 variants were considered (listed in Table 1). Canonical forms of core histones were included in the analysis. Multiple sequence alignments of the selected histones were performed using Clustal Omega on the EMBL-EBI Web site [39], from which the percentage of similarity between sequences was downloaded and processed with R-Studio to create the plots displayed in Fig. 2.

### Histone extraction from mouse testes and digestion for their MS analysis

Histones were extracted as previously described [14]. Suspensions of whole testis cells were obtained from mice older than 2 months. Enriched fractions of spermatocytes, round spermatids, and elongated spermatids were obtained as described previously [62, 72].

The purification of histones was obtained by resuspending washed cell pellets in 0.2 M sulfuric acid. The solution was sonicated to shear DNA and was placed on ice for 30 min. Non-soluble proteins were pelleted by 15-min centrifugation at 20,000*g*. The supernatant with solubilized histones was collected and proteins were precipitated with Trichloroacetic acid (TCA) 20% v/v. After 90-min incubation on ice, proteins were pelleted by 15-min centrifugation at 16,000*g*. They were then washed with acidified acetone (HCl 0.1%) and then with pure acetone. Pellets were air-dried at room temperature and resuspended in 1× protein loading buffer. The quality of the purification was then analyzed by Coomassie-stained SDS–PAGE gels.

For targeted proteomic analyses, histones were only migrated over about 5 mm in the stacking region of the gel, before reduction, alkylation of Cys residues with iodoacetamide and trypsin digestion, as described previously [73]. For the in-depth search for PTMs on signature peptides, histone samples were fully migrated, seven gel slices corresponding to histones were cut, individually digested and analyzed by discovery LC–MS/MS using an UltiMate 3000 system coupled to a Q-Exactive HF instrument (Thermo Fisher Scientific): The lower complexity of the resulting samples indeed allowed more exhaustive characterization of the modified peptides.

### Analysis of standard peptides to build a spectral library for SRM analysis

Forty-six synthetic peptides of “crude quality” with a C-terminal [^13^C,^15^N]- labeled lysine, arginine, alanine, or tyrosine were purchased from Thermo Fisher Scientific. They were pooled at an estimated concentration of 0.029–321 pmol/μL (Additional file 6), due to differences in ionization efficiency. By spiking the labeled peptides at these concentrations in histone samples extracted from mouse testis, their signal intensity was close to the endogenous peptides. The labeled peptide mixture was subjected to LC–MS/MS analyses on a C18 column (PepMap C18, 100 Å porosity, 3 μm particles, 25 cm length × 75 μm inner diameter) coupled to a QTRAP 5500 or an LTQ-Orbitrap Velos system to constitute a spectral library. Peptides were separated at a flow rate of 300 nL/min with a gradient starting with solvent A = acetonitrile/formic acid/water (2/0.1/97.9, v/v/v), then developing 0–40% of solvent B = acetonitrile/formic acid/water (80/0.08/19.92, v/v/v) over 35 min, followed by 40–90% solvent B over 10 min and maintaining 90% solvent B for 10 min. Based on these LC–MS/MS analyses, transitions were selected and validated for LC-SRM analysis. Quality control samples (cytochrome c, GFP) were analyzed at the beginning and at the end of a series of injections to verify instrument performances.

### SRM analyses of histones spiked with standards on the Qtrap instrument

Two biological samples were analyzed in three technical replicates, where technical replicates started at the time of histone loading on an SDS–PAGE gel. SRM measurements were performed on a Qtrap mass spectrometer (QTrap 5500, Sciex). For each analysis, the equivalent of about 1/50 of the digested histones extracted from 4 million spermatocytes, 5 million round spermatids, or 10 million condensing/elongating spermatids was analyzed. Tryptic peptides were separated on an analytical column with C18 Pepmap beads (3 μm diameter, 100 Å porosity, 25 cm length × 75 μm inner diameter), at a flow rate of 300 nL/min. Peptides were separated with the same gradient as the one described in the section “*Peptides selection and synthesis*.” The SRM analyses were performed with a dwell time of 30 ms, a retention time window of 5 min, and a fixed cycle time of 3 s.

The linearity curves were obtained by preparing, in triplicate, a dilution series (0.01; 0.03; 0.1; 0.25; 0.5; 0.75; 1) of the labeled peptide mixture or of the biological matrix into a constant amount of mouse testis histone extract or a constant amount of labeled peptide mixture, respectively. To each diluted sample, 2 μL of either the set of 46 peptides or biological matrix were spiked to make up to 10 μL total sample volume. The samples were transferred to injection vials, and 6 μL were injected on the LC-SRM system.

### PRM analyses of histones on a Q-Exactive Instrument

PRM analyses were performed using a Q-Exactive hybrid quadrupole-orbitrap mass spectrometer (Thermo Fisher Scientific). The UltiMate 3000 HPLC system was equipped with a capillary column containing ReproSil-Pur C18-AQ beads (1.9 μm, 25 cm length, 75 μm inner diameter). The acquisition method consisted of acquiring one MS spectrum and 10 PRM spectra. The same LC gradient as for LC-SRM analyses was used. For PRM spectra, a target resolution of 60,000, an automatic gain control (AGC) value of 5.5 × 10^5^, and a maximum injection time of 100 ms were specified. Fragmentation was performed with a normalized collision energy of 27, and MS/MS scans were acquired with a starting mass of m/z 100.

### Discovery LC–MS/MS data interpretation

Mass spectrometry RAW files were submitted to Mascot Daemon (version 2.5.1). MS/MS data acquired on histones were matched to the mouse MS_HistoneDB [8] and to a list of about 500 contaminants including keratins, trypsin, etc. The following modifications were considered as variable ones: N-terminal protein acetylation; Lys acetylation; Lys and Arg mono- and dimethylations, Ser and Thr phosphorylation, Met oxidation. Cys carbamydomethyl was considered as a fixed modification. For RAW files of full MS/MS spectra acquired on the Qtrap and LTQ-Orbitrap Velos instruments, the tolerance on mass measurement was set to 5 ppm for peptides and to 0.8 Da (Qtrap) and 0.6 Da (LTQ-Orbitrap Velos) for fragment ions. For analysis performed on The Q-Exactive instrument, 5 ppm for peptides and 0.025 Da for fragment ions were considered. For all MS/MS data interpretations using Mascot, the enzyme trypsin was specified, while allowing up to five tryptic missed cleavages.

### Selection of SRM transitions from experimental LC–MS/MS data

A spectral library was built in the academic open-source software Skyline [74]. MS/MS data used for that purpose had been obtained on histones extracted from mouse testis and digested by trypsin, as well as from standard peptides, analyzed on Qtrap and LTQ-Orbitrap instruments (see analyses conditions described above). We then selected the best responding fragments ranked by intensity. We globally selected y-type fragment ions, and in particular those ending with a Proline residue. In SRM analyses using heavy-isotope-labeled sequences, only y-type ions were used for quantification.

### Targeted proteomics data interpretation

The acquired SRM and PRM data were processed using Skyline 2.6. Transitions selected for each peptide in SRM were used as quantifiers (the process of transition selection is presented in Additional file 9). They were manually integrated based on the chromatographic traces extracted by the program, so as to avoid possible co-eluting contaminants. Both for PRM and SRM data, the transition selection was systematically verified and adjusted when necessary to ensure that no co-eluting contaminant distorted quantification. The visual filtering was performed as follows: (1) selection of peaks presenting an intensity five times higher than the noise signal; (2) verification that near-identical relative intensities were observed for the transitions of the endogenous and of the standard peptides. When these criteria were met by no transition, no quantification values were reported.

### Data normalization to be at constant nucleosome amounts in the compared samples

The abundance of peptides at different stages of spermatogenesis was usually normalized by the sum of H4 peptides (P50–52 in Table 1). However, this normalization was not adapted when comparing the expression levels of histone variants extracted from round spermatids of WT or Sly-KD mice. Indeed, the purity of the round spermatids was different in the two biological replicates, even though it was above 84%. The contamination by spermatocytes could profoundly impact the histone variant quantification in round spermatids, since spermatocytes contain about four times more chromatin than round spermatids. We then only considered variants that are specifically expressed in spermatids for normalization: we used peptide P29 shared by H2A.L.2 and Y-ChrH2A.L.2, whose abundance was not expected to be affected by knocking down sly.

## Additional files

**Additional file 1.** Sequence analysis of histone H3 variants. An in silico analysis predicts that only five peptides discriminating H3 variants could be followed by mass spectrometry (these peptides are highlighted in blue boxes). None of them passed the filters used to develop the SRM assay on H2A and H2B variants. For more details, please refer to the section “Theoretical histone peptides relevant for a targeted proteomic analysis” of the "Methods" section.

**Additional file 2.** Validation criteria for each signature peptide. Peptide number, protein names, peptide sequence, ESP predictor score, and length of peptides are indicated. The criteria detailed in the results sections (*Theoretical histone peptides relevant for a targeted proteomic analysis* and *Trypsin missed cleavages in signature peptides)* were also reported for each peptide. They include their status as fully tryptic or non-tryptic (e.g., C-terminal of the histone variant), if their length is within 6–23 amino acids, if they are not chemically modified, and if the monitored peptides have no neighboring K/R residue.

**Additional file 3.** Abundance of H4 peptides used for normalization. The nucleosome contains two copies of each histone. No H4 variant has been described in mammals [8], so that H4 was logically chosen to normalize the abundance of H2A and H2B variants between samples. The MS signals for three H4 peptides (P50–52) are presented for the analysis of histones extracted from spermatocytes (Sc), round spermatids (R), and elongating and condensing spermatids (EC). They were brought to the same scale as for spermatocytes to allow easier inter-peptide signal comparisons. Three technical replicates of LC–MS/MS analyses were analyzed. The relative MS signals measured on peptides P50–P52 are similar in the three cell types.

**Additional file 4.** Details of the SRM transitions for each signature peptide. SRM assay parameters including precursor and fragment ion type, charge state, elution time as well as raw data are provided in Suppl. data. (*) Indicates peptides monitored only in their endogenous form.

**Additional file 5.** Composition of the mixture of standard peptides.

**Additional file 6.** Reproducibility of the LC-SRM analysis. R² values are indicated for each technical replicate with increasing matrix amounts or increasing standard peptide amounts. Plotted data are presented in Fig. 5.

**Additional file 7.** PRM transition results. List of transitions used to quantify each monitored peptide. Precursor charge, fragment ion, light precursor m/z ratio, light product m/z ratio, light retention time are indicated.

**Additional file 8.** Abundances of modified and non-modified forms of H2B and H4 signature peptides in round spermatids extracted from WT and Sly-KD mice testis. Two biological replicates are presented.

**Additional file 9.** Rules used to select or reject peptides using their transition profiles. The validation of the best transitions was performed using a signal-to-noise ratio (> 5) and a perfect co-elution of the heavy standard peptide with the endogenous peptide. Three fragment ions (F1, F2, and F3) are represented for the heavy and the endogenous peptides. **a** All fragment ions can be integrated because the heavy and endogenous fragment ions co-elute in the same intensity order. **b** In that case, only F2 can be integrated because the ratio heavy/endogenous is different for F1 and F3. **c** The fragment F2 is contaminated by another analyte eluting at a slightly later time; it has to be excluded from the analysis. **d** Here, the signal-to-noise ratio is below five, no fragment ion can be integrated. **e.** The endogenous peptide traces do not co-elute with the heavy peptide traces.

## Abbreviations

CID: collision-induced dissociation
EC: elongating and condensing spermatids
ESI: electrospray ionization
HPLC: high-pressure liquid chromatography
MS: mass spectrometry
MS/MS: fragmentation by tandem mass spectrometry
PRM: parallel reaction monitoring
PTM: post‐translational modification
R: round spermatids
RT: retention time
Sc: spermatocytes
SID: stable isotope dilution
SRM: selected reaction monitoring
QQQ: triple quadrupole
q-OT: hybrid quadrupole-orbitrap
