# Supplementary figures and images for: Systematic quantitative analysis of H2A and H2B variants by targeted proteomics
Source: Epigenetics Chromatin. 2018 Jan 12;11:2. doi: 10.1186/s13072-017-0172-y (PMC5767011; doi:10.1186/s13072-017-0172-y)

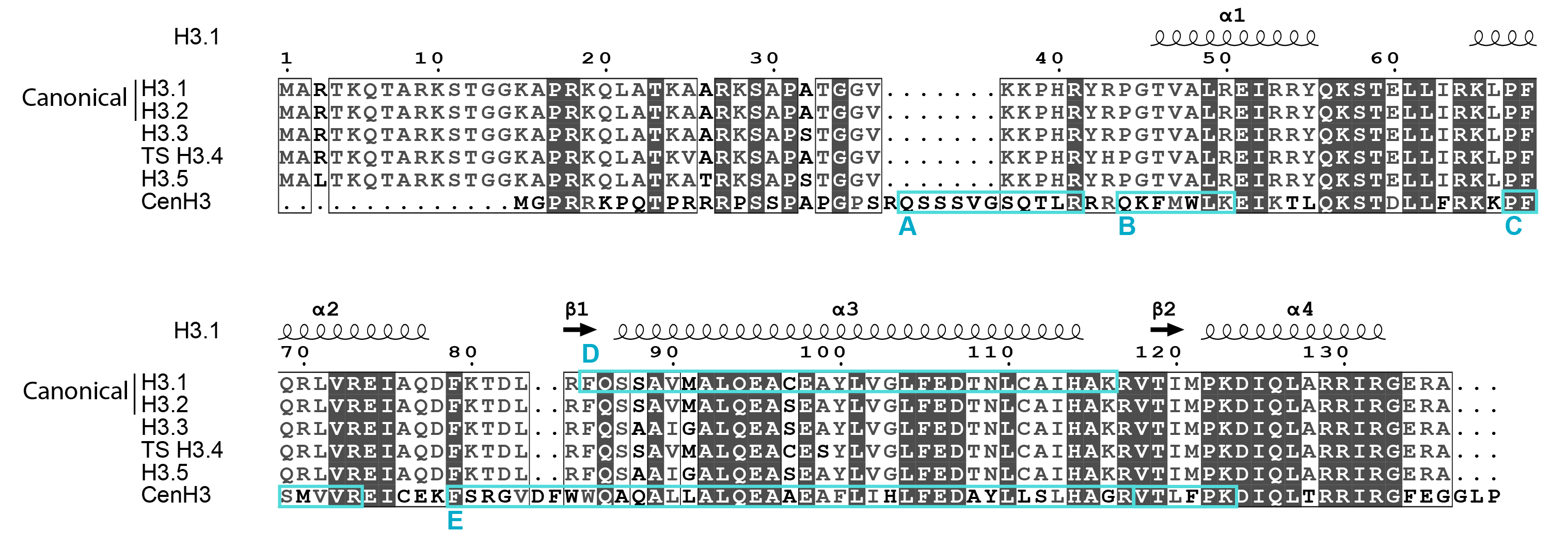

Supplement: Supplementary file 1 — Additional file 1. Sequence analysis of histone H3 variants. An in silico analysis predicts that only five peptides discriminating H3 variants could be followed by mass spectrometry (these peptides are highlighted in blue boxes). None of them passed the filters used to develop the SRM assay on H2A and H2B variants. For more details, please refer to the section “Theoretical histone peptides relevant for a targeted proteomic analysis” of the "Methods" section. [file 13072_2017_172_MOESM1_ESM.png]

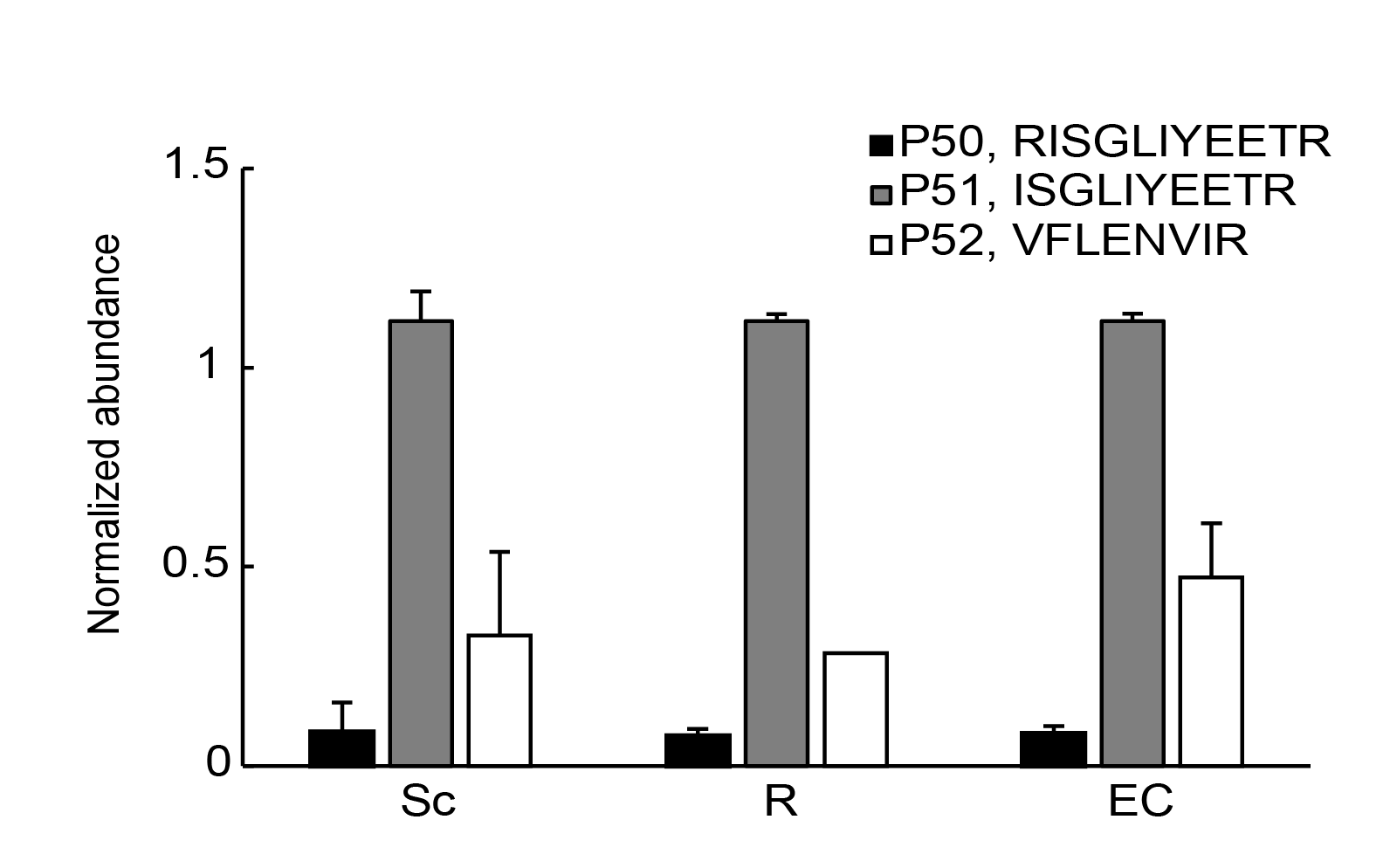

Supplement: Supplementary file 3 — Additional file 3. Abundance of H4 peptides used for normalization. The nucleosome contains two copies of each histone. No H4 variant has been described in mammals [8], so that H4 was logically chosen to normalize the abundance of H2A and H2B variants between samples. The MS signals for three H4 peptides (P50–52) are presented for the analysis of histones extracted from spermatocytes (Sc), round spermatids (R), and elongating and condensing spermatids (EC). They were brought to the same scale as for spermatocytes to allow easier inter-peptide signal comparisons. Three technical replicates of LC–MS/MS analyses were analyzed. The relative MS signals measured on peptides P50–P52 are similar in the three cell types. [file 13072_2017_172_MOESM3_ESM.png]

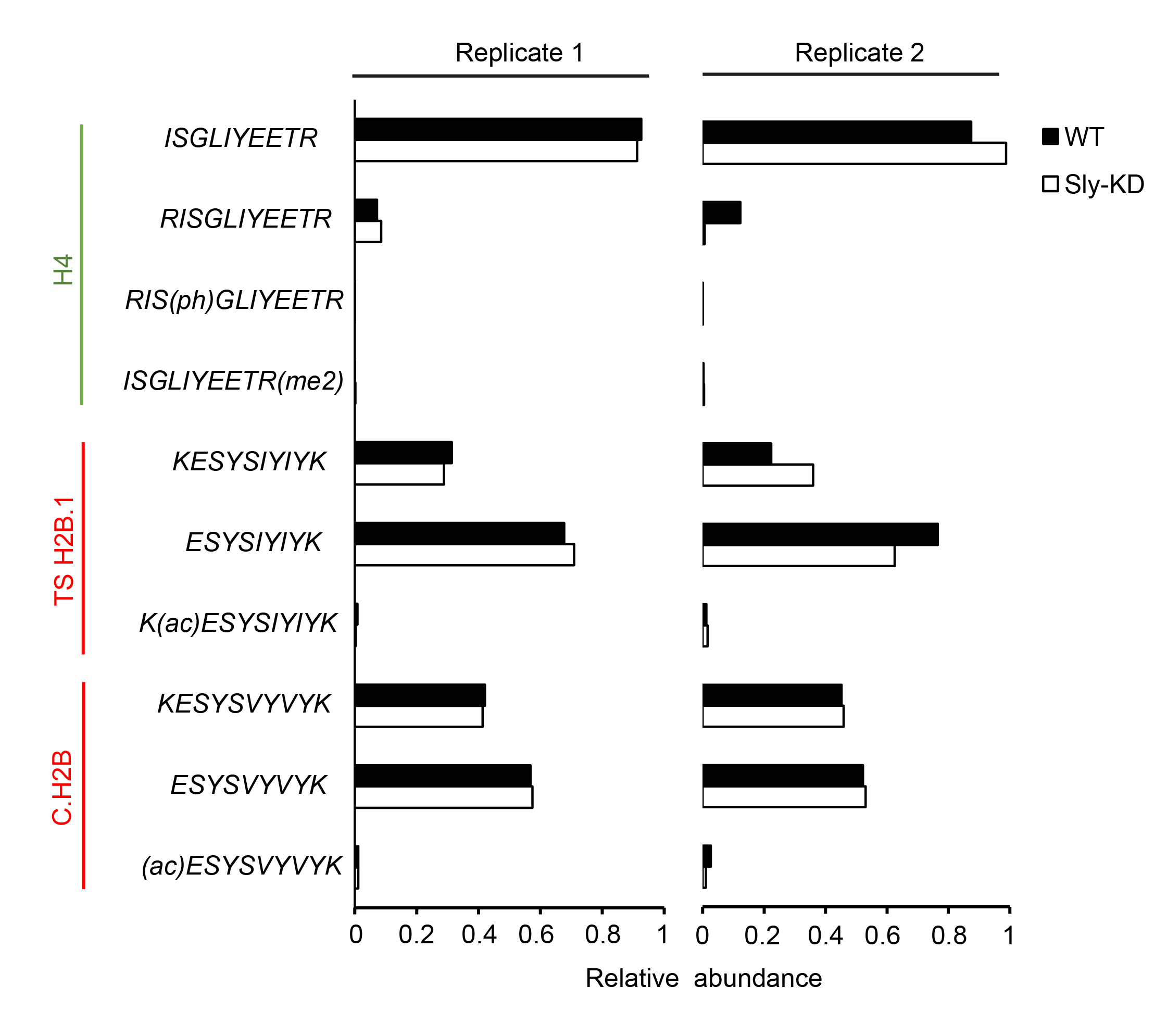

Supplement: Supplementary file 8 — Additional file 8. Abundances of modified and non-modified forms of H2B and H4 signature peptides in round spermatids extracted from WT and Sly-KD mice testis. Two biological replicates are presented. [file 13072_2017_172_MOESM8_ESM.png]

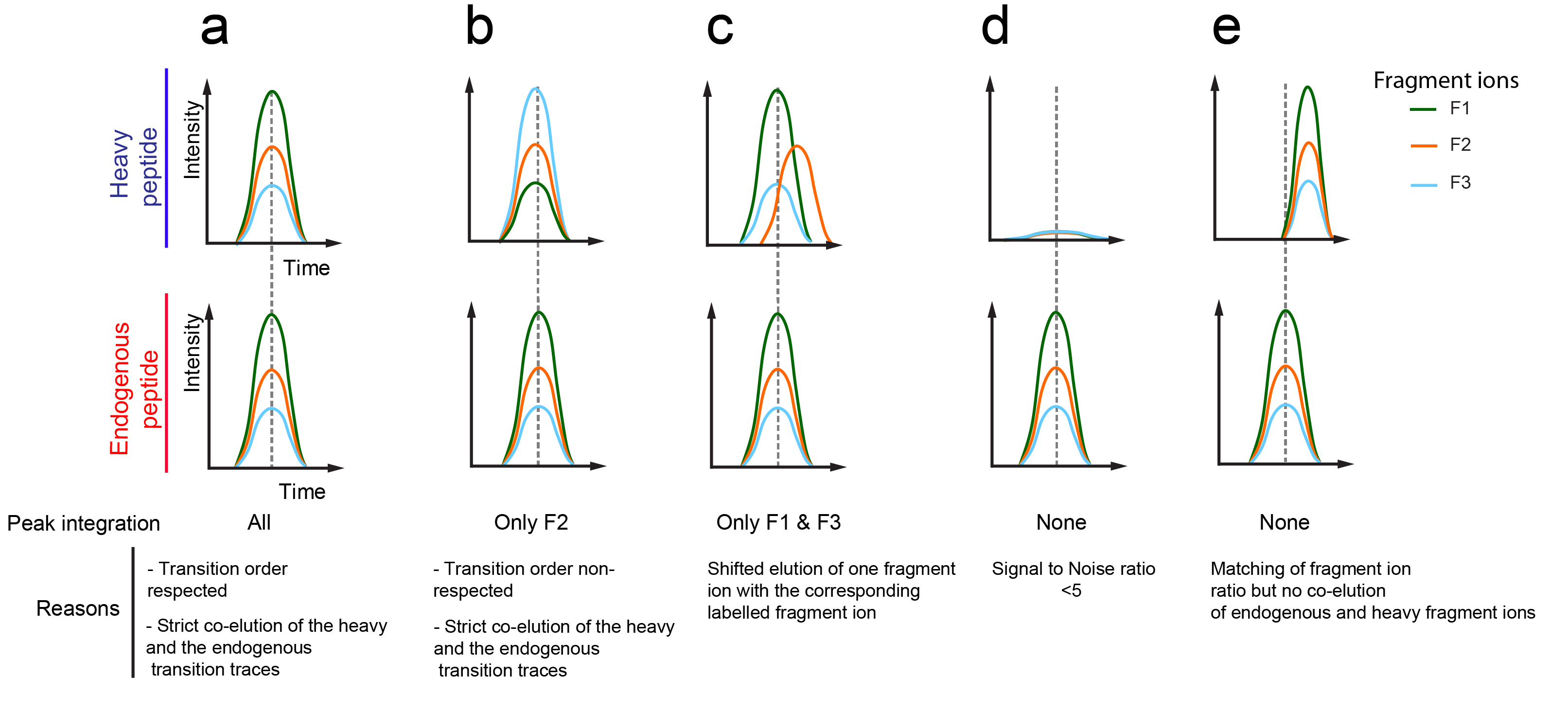

Supplement: Supplementary file 9 — Additional file 9. Rules used to select or reject peptides using their transition profiles. The validation of the best transitions was performed using a signal-to-noise ratio (> 5) and a perfect co-elution of the heavy standard peptide with the endogenous peptide. Three fragment ions (F1, F2, and F3) are represented for the heavy and the endogenous peptides. a All fragment ions can be integrated because the heavy and endogenous fragment ions co-elute in the same intensity order. b In that case, only F2 can be integrated because the ratio heavy/endogenous is different for F1 and F3. c The fragment F2 is contaminated by another analyte eluting at a slightly later time; it has to be excluded from the analysis. d Here, the signal-to-noise ratio is below five, no fragment ion can be integrated. e. The endogenous peptide traces do not co-elute with the heavy peptide traces. [file 13072_2017_172_MOESM9_ESM.png]
