# Supplementary material for: Systematic quantitative analysis of H2A and H2B variants by targeted proteomics
Source: Epigenetics Chromatin. 2018 Jan 12;11:2. doi: 10.1186/s13072-017-0172-y (PMC5767011; doi:10.1186/s13072-017-0172-y)
Supplement: Supplementary file 5 — Additional file 5. Composition of the mixture of standard peptides. [file 13072_2017_172_MOESM5_ESM.docx]

**Additional file 6: Table S3. Composition of the mixture of standard peptides**.

| N°= | Peptides | Proteins | Molecular weight | ESP predictor score | [Stock] µg/µL | Standard spiked pmol/uL |
| --- | --- | --- | --- | --- | --- | --- |
| 1 | AGLQFPVGR | Canonical H2A, H2A.J, H2A.X, H2AZ.1, H2AZ.2, TS H2A.1 | 955 | 0.739 | 1 | 0.045 |
| 3 | ASQASQEY | H2A.X | 896 | 0.278 | 13.5 | 321.000 |
| 4 | GDEELDSLIK | H2AZ.1, H2AZ.2 | 1126 | 0.519 | 2 | 0.076 |
| 5 | ATIAGGGVIPHIHK | H2AZ.1, H2AZ.2 | 1379 | 0.522 | 3.25 | 5.020 |
| 6 | AGVIFPVGR | Macro-H2A.1, Macro-H2A.2, Macro-H2A.3 | 925 | 0.575 | 5 | 0.115 |
| 7 | HILLAVANDEELNQLLK | Macro-H2A.1, Macro-H2A.2 | 1941 | 0.222 | 1.25 | 13.700 |
| 8 | LEAIITPPPAK | Macro-H2A.1 | 1157 | 0.713 | 5.25 | 0.064 |
| 9 | AASADSTTEGTPTDGFTVLSTK | Macro-H2A.1 | 2165 | 0.517 | 2.5 | 24.600 |
| 10 | NGPLEVAGAAISAGHGLPAK | Macro-H2A.1 | 1838 | 0.723 | 7.75 | 8.970 |
| 11 | SIAFPSIGSGR | Macro-H2A.1 | 1102 | 0.796 | 0.75 | 0.029 |
| 12 | GVTIASGGVLPR | Macro-H2A.2, Macro-H2A.3 | 1137 | 0.866 | 1.5 | 0.056 |
| 13 | SETILSPPPEK | Macro-H2A.2 | 1205 | 0.58 | 5.75 | 0.203 |
| 14 | EGTSNSTSEDGPGDGFTILSSK | Macro-H2A.2 | 2194 | 0.499 | 11.75 | 11.400 |
| 15 | SVAFPPFPSGR | Macro-H2A.2 | 1171 | 0.578 | 6.75 | 0.123 |
| 16 | NCLSAAEIR | Macro-H2A.3 | 986 | 0.726 | 4.25 | 0.183 |
| 17 | SPVAETASPGRPGDPQGHLGSLR | Macro-H2A.3 | 2296 | 0.664 | 5.5 | 51.000 |
| 18 | AGDGQTGHQVALSGSGGEGGSA | Macro-H2A.3 | 1904 | 0.504 | 3.75 | 41.900 |
| 19 | QGNYAQR | TS H2A.1 | 846 | 0.089 | 4.75 | 119.000 |
| 20 | GELPFSLVDR | H2A.L.1- *H2al1a* | 1142 | 0.821 | 6.75 | 0.251 |
| 21 | IAPEDVR | H2A.L.1- *H2al1a, H2al1e, H2al1k, H2al1o* | 809 | 0.299 | 4.75 | 0.250 |
| 22 | GELPLSLVDR | H2A.L.1- *H2al1b, H2al1e, H2al1k, H2al1m, H2al1n* | 1108 | 0.814 | 5.5 | 0.211 |
| 23 | IAPEDVHLVVQNNEQLR | H2A.L.1- *H2al1b* | 1984 | 0.301 | 8.25 | 8.850 |
| 24 | LVVQNNEQLR | H2A.L.1- *H2al1a, H2al1e, H2al1k, H2al1m* | 1222 | 0.596 | 6 | 0.209 |
| 25 | GEFPLSLVDR | H2A.L.1- *H2al1j* | 1142 | 0.821 | 7.25 | 0.270 |
| 26 | FLPEGNHSGR | H2A.L.1- *H2al1j* | 1123 | 0.445 | 5.75 | 109.000 |
| 27 | VTPEDVR | H2A.L.1- *H2al1m* | 825 | 0.216 | 5.25 | 13.500 |
| 28 | GELPLSLVDHFLR | H2A.L.1- *H2al1o* | 1506 | 0.262 | 8 | 11.300 |
| 29 | AELQFPVSR | H2A.L.2, Y-Chr H2A.L.3 | 1057 | 0.844 | 1.25 | 0.050 |
| 31 | IAPEHVCR | H2A.L.2 | 934 | 0.438 | 5.5 | 125.000 |
| 33 | FLGEGIYSR | Y-Chr H2A.L.3 | 1051 | 0.433 | 5.75 | 0.116 |
| 34 | IAPEHVCQVVQNK | Y-Chr H2A.L.3 | 1473 | 0.464 | 5 | 0.144 |
| 35 | NAPFSLFDEMPGPR | H2A.P | 1588 | 0.673 | 7 | 0.188 |
| 36 | NTENCLQR | H2A.B.2 | 987 | 0.26112 | 3.75 | 8.080 |
| 37 | LLELAGNEAQR | H2A.B.2, H2A.B.3 | 1223 | 0.77676 | 5 | 0.058 |
| 38 | KESYSVYVYK | Canonical H2B | 1273 | 0.227 | 5.25 | 8.770 |
| 39 | ESYSVYVYK | Canonical H2B | 1145 | 0.359 | 1.75 | 0.065 |
| 40 | EIQTAVR | Canonical H2B, TS H2B.1 | 827 | 0.331 | 0.5 | 1.290 |
| 41 | LLLPGELAK | Canonical H2B, TS H2B.1 | 961 | 0.517 | 4.75 | 0.210 |
| 42 | KESYSIYIYK | TS H2B.1 | 1301 | 0.227 | 4.5 | 0.147 |
| 43 | ESYSIYIYK | TS H2B.1 | 1173 | 0.311 | 4.75 | 0.172 |
| 44 | KLATLAVTFGSK | subH2B | 1243,43 | 0.608 | 5.75 | 9.840 |
| 45 | LATLAVTFGSK | subH2B | 1115,25 | 0.571 | 4.5 | 0.172 |
| 46 | NSFAIYFPK | H2B.L.2 | 1094,19 | 0.398 | 6 | 0.117 |
| 47 | SVNILDSFVK | H2B.L.2 | 1129,23 | 0.421 | 5.25 | 0.198 |
| 48 | IASEASFLAR | H2B.L.2 | 1074,13 | 0.728 | 5.75 | 0.076 |
