# Supplementary material for: Systematic quantitative analysis of H2A and H2B variants by targeted proteomics
Source: Epigenetics Chromatin. 2018 Jan 12;11:2. doi: 10.1186/s13072-017-0172-y (PMC5767011; doi:10.1186/s13072-017-0172-y)
Supplement: Supplementary file 6 — Additional file 6. Reproducibility of the LC-SRM analysis. R² values are indicated for each technical replicate with increasing matrix amounts or increasing standard peptide amounts. Plotted data are presented in Fig. 5. [file 13072_2017_172_MOESM6_ESM.docx]

**Additional file 5: Table S2. Reproducibility of the LC-SRM analysis.**

R² values are indicated for each technical replicate with increasing matrix amounts or increasing standard peptide amounts. Plotted data are presented in Figure 5.

|  |  |  | Endogenous to standard R ² | | | | Standard to endogenous R² | | | |
| --- | --- | --- | --- | --- | --- | --- | --- | --- | --- | --- |
|  | Protein | name | Rep1 | Rep2 | Rep3 | Range of measured dilution factors | Rep1 | Rep2 | Rep3 | Range of measured dilution factors |
| 4 | H2A.Z.1/2 | GDEELDSLIK | 0.993 | 0.985 | 0.972 | 0.03-1 | 0.999 | 0.994 | 0.984 | 0.03-1 |
| 5 | H2A.Z.1/2 | ATIAGGGVIPHIHK | 0.988 | 0.994 | 0.990 | 0.01-1 | 0.992 | 0.989 | 0.946 | 0.01-1 |
| 6 | Macro-H2A.1/2/3 | AGVIFPVGR | 0.997 | 0.994 | 0.997 | 0.03-1 | 0.989 | 0.997 | 0.998 | 0.01-1 |
| 11 | Macro-H2A.1 | SIAFPSIGSGR | 0.997 | 0.947 | 0.984 | 0.03-1 | 0.998 | 0.993 | 0.996 | 0.01-1 |
| 13 | Macro-H2A.1/2 | GVTIASGGVLPR | 0.997 | 0.962 | 0.994 | 0.1-1 | NA | NA | NA | 0.01-1 |
| 20 | H2A.L.1- *H2al1a* | GELPFSLVDR | 0.994 | 0.964 | 0.986 | 0.1-1 | 0.982 | 0.997 | 0.978 | 0.01-1 |
| 22 | H2A.L.1-*H2al1b,H2al1e,H2al1k,H2al1m* | GELPLSLVDR | 0.997 | 0.971 | 0.989 | 0.01-1 | 0.995 | 0.993 | 0.971 | 0.01-1 |
| 24 | H2A.L.1-*H2al1a,H2al1e,H2al1k,H2al1m* | LVVQNNEQLR | 0.998 | 0.995 | 0.992 | 0.1-1 | 0.983 | 0.975 | 0.995 | 0.01-1 |
| 29 | H2A.L.2; Y-chr H2A.L.3 | AELQFPVSR | 0.995 | 0.971 | 0.990 | 0.01-1 | 0.999 | 0.997 | 0.990 | 0.03-1 |
| 38 | canonical H2B | KESYSVYVYK | NA | NA | NA | 0.01-1 | 0.978 | 0.990 | 0.985 | 0.01-1 |
| 39 | canonical H2B | ESYSVYVYK | 0.993 | 0.990 | 0.991 | 0.01-1 | 0.994 | 1.000 | 0.980 | 0.01-1 |
| 41 | canonical H2B; TS H2B.1 | LLLPGELAK | 0.993 | 0.992 | 0.996 | 0.01-1 | 0.997 | 0.993 | 0.990 | 0.01-1 |
| 42 | canonical H2B; TS H2B.1 | KESYSIYIYK | 0.966 | 0.968 | 0.982 | 0.01-1 | 0.973 | 0.969 | 0.962 | 0.01-1 |
| 43 | TS H2B.1 | ESYSIYIYK | 0.990 | 0.969 | 0.978 | 0.01-1 | 0.965 | 0.982 | 0.950 | 0.01-1 |
